# Evolutionary dynamics of origin and loss in the deep history of phospholipase D toxin genes

**DOI:** 10.1186/s12862-018-1302-2

**Published:** 2018-12-18

**Authors:** Matthew H. J. Cordes, Greta J. Binford

**Affiliations:** 1 Department of Chemistry and Biochemistry, University of Arizona, Tucson, AZ 85701 USA; 2 Department of Biology, Lewis & Clark College, Portland, OR 97219 USA

**Keywords:** Spider venom, Molecular evolution, Lateral gene transfer, Phospholipase D, Gene loss

## Abstract

**Background:**

Venom-expressed sphingomyelinase D/phospholipase D (SMase D/PLD) enzymes evolved from the ubiquitous glycerophosphoryl diester phosphodiesterases (GDPD). Expression of GDPD-like SMaseD/PLD toxins in both arachnids and bacteria has inspired consideration of the relative contributions of lateral gene transfer and convergent recruitment in the evolutionary history of this lineage. Previous work recognized two distinct lineages, a SicTox-like (ST-like) clade including the arachnid toxins, and an Actinobacterial-toxin like (AT-like) clade including the bacterial toxins and numerous fungal homologs.

**Results:**

Here we expand taxon sampling by homology detection to discover new GDPD-like SMase D/PLD homologs. The ST-like clade now includes homologs in a wider variety of arthropods along with a sister group in Cnidaria; the AT-like clade now includes additional fungal phyla and proteobacterial homologs; and we report a third clade expressed in diverse aquatic metazoan taxa, a few single-celled eukaryotes, and a few aquatic proteobacteria. GDPD-like SMaseD/PLDs have an ancient presence in chelicerates within the ST-like family and ctenophores within the Aquatic family. A rooted phylogenetic tree shows that the three clades derived from a basal paraphyletic group of proteobacterial GDPD-like SMase D/PLDs, some of which are on mobile genetic elements. GDPD-like SMase D/PLDs share a signature C-terminal motif and a shortened βα1 loop, features that distinguish them from GDPDs. The three major clades also have active site loop signatures that distinguish them from GDPDs and from each other. Analysis of molecular phylogenies with respect to organismal relationships reveals a dynamic evolutionary history including both lateral gene transfer and gene duplication/loss.

**Conclusions:**

The GDPD-like SMaseD/PLD enzymes derive from a single ancient ancestor, likely proteobacterial, and radiated into diverse organismal lineages at least in part through lateral gene transfer.

**Electronic supplementary material:**

The online version of this article (10.1186/s12862-018-1302-2) contains supplementary material, which is available to authorized users.

## Background

Comparative evolutionary analyses provide insight into origin of novel function, as well as the dynamics and directionality of phenotypic change. As new sequence data become available, we can better assess the relative importance of convergence, including independent recruitment from widespread gene families, and lateral gene transfer (LGT) in the origin of novel phenotypes. The evolutionary history of phospholipase D (PLD) toxins is a particularly interesting case study. Emerging understanding of the phylogenetic distribution of these toxins has inspired hypotheses about roles of lateral gene transfer and/or convergent recruitment of toxic activity, and the identification of functionally relevant motifs [1–3]. The recognition of similarities among disparate lineages that express these toxins has potential to elucidate common features of the toxic effects on mammals, facilitate development of widespread treatments, and illuminate general cascades of human pathophysiological response.

PLD toxins are well known from a variety of pathogenic organisms, most famously in venoms of brown recluse spiders and their relatives. Comparative evidence supports recruitment of the toxin in spider venoms before the most recent common ancestor of sicariid spiders, which include the genera *Loxosceles, Sicarius,* and *Hexophthalma* (formerly African *Sicarius*) [4, 5]. We therefore have referred to the spider toxin gene family as *SicTox* [4]. Multiple paralogs of *SicTox* are highly expressed in venom and serve functional roles in prey immobilization [6], but these enzymes are most notorious for being sufficient to cause severe dermonecrotic lesions, and occasional systemic effects, in mammals [7]. The enzyme activity is also functionally relevant in tick saliva (*Ixodes*) [8], venom of a buthid scorpion [9], and as exotoxins in two genera of pathogenic bacteria [10]. Homologs with less well-described function are also in pathogenic fungi [2, 3]. Felicori and coworkers [2] recently expanded the known distribution of homologs to include: 11 genera in four orders of fungi; spider taxa with sequenced genomes (Araneomorph *Stegodyphus* & mygalomorph *Acanthoscurria*), two new genera of ixodine ticks, predatory mites (*Metaseiulus*) and dust house mites (*Dermatophagiodes*); two new suborders of Actinomycetales bacteria (*Streptomyces, Austwickia*) and a genus of proteobacteria in the order Burkholderiales (*Burkholderia*). A phylogenetic analysis supported two major clades, a SicTox-like (ST-like) clade that includes the arachnid toxins, and an Actinobacterial-toxin like (AT-like) clade that includes the bacterial toxins as well as fungal homologs, with the *Burkholderia* homolog a singleton outside of both broader groups [2].

Phospholipase D toxins belong to a protein domain family known as the GDPD-like SMase D/PLDs (NCBI conserved protein domain family cd08576). This family is unrelated to the better-known HKD PLDs [11], and instead evolved from glycerophosphoryl diester phosphodiesterase (GDPD), a ubiquitous gene family that hydrolyzes glycerol phosphodiesters and plays a role in glycerol metabolism [1, 12, 13]. PLD toxins were originally described as sphingomyelinase D enzymes (SMase D), [14] but later studies showed some to be broader spectrum PLDs capable of acting on diverse lysophospholipids [15, 16], as well as other phosphosphingolipids [17]. The name GDPD-like SMase/PLD thus reflects both evolutionary origin and ambiguous substrate preference. While the most well-characterized members of this family are toxins, some members of the family may not be; for example, there are spider homologs expressed in non-venom tissues that are serving some other function within the organism (Binford, unpublished).

PLD toxins have retained some shared or overlapping features with GDPD relatives while also diverging considerably. Key active site residues are conserved between the two groups [1]. GDPDs act primarily on glycerophosphodiester substrates, and PLD toxins are best known as sphingomyelinases, but at least a few GDPDs and many GDPD-like SMaseD/PLDs have lysophospholipase activity [15, 16, 18–20]. PLD toxins are generally extracellular enzymes [21, 22], while only a subset of GDPD family members are secreted [18]. All known members of the PLD toxin lineage have a characteristic C-terminal motif, proposed to increase structural stability [2, 4]. This motif is lacking in GDPD domains, and is thus a shared, derived character, or synapomorphy, for the GDPD-like SMaseD/PLD clade [1]. Finally, GDPD enzymes catalyze a two-step reaction in which an alcohol group on the substrate is displaced by intramolecular attack of a hydroxyl group, generating a cyclic intermediate, which is hydrolyzed in a second step [23]. The mechanism of PLD toxins from bacteria, fungi and spiders lacks the hydrolytic step, so that the cyclic intermediate is the final product [19, 24].

The disparate phylogenetic distribution of these enzymes, coupled with their common structural motifs and lack of hydrolytic chemistry, has fueled the question of how the taxonomic distribution of PLD toxins arose. Is it a product of convergent evolution from different GDPD ancestors, or of divergent evolution accompanied by lateral gene transfer [1–3]? We initially argued the case for lateral gene transfer based on the evidence of the synapomorphy of the C-terminal motif shared between bacterial and spider SMase D [1]. With the detection of homologs in fungi, Fry et al. argued that the gene family was likely widespread among Opisthokonts and convergently recruited for antagonistic function in fungi and spiders [3]. With a further expanded data set, Felicori and coworkers have suggested that lateral transfer between metazoans and bacteria is likely, but also that the recruitment of the secreted toxin may have occurred convergently within fungi and arachnids [2]. The discovery that bacterial, fungal and spider PLD toxins catalyze cyclization (not hydrolysis) led us to reassert our original hypothesis of divergence plus lateral gene transfer [19].

Here we use homology searching and phylogenetic analysis to probe the deeper evolutionary history of PLD toxins in the GDPD-like SMaseD/PLD family. Through updated sequence similarity searching, we discover a new lineage of proteins related to PLD toxins, found in a diverse array of aquatic organisms. We infer that the ST-like homologs (which include the spider toxins), the AT-like homologs (which include the actinobacterial toxins and fungal homologs) and the new mainly aquatic lineage comprise three major clades. These three clades diverged from a basal paraphyletic group that likely originated in proteobacteria. We identify multiple molecular synapomorphies indicating that all GDPD-like SMaseD/PLD family members diverged from a single ancestor, probably bacterial. Finally, we find that lateral gene transfer, but also gene duplication and loss, contributed to the unusual species distribution of PLD toxins.

## Results

### Sequence similarity searching

We conducted initial protein BLAST searches using three representatives of the SicTox-like (ST-like) family and three from the Actinobacterial-toxin like (AT-like) family as query sequences, along with a singleton sequence from the proteobacterium *Burkholderia*. We accepted hits with E < 1e-05 (E-value) to a single query sequence or E < 1e-03 to at least two query sequences. These initial searches identified PLD-toxin homologs in previously unrepresented lineages (Additional file 1: Table S1). For ST queries, these included other arthropod classes and subphyla such as the Merostomata and crustaceans, but also distantly related organisms such as cnidarians and proteobacteria. For AT and *Burkholderia* queries these included other fungal phyla such as the basidiomycota, but also oceanic diatoms and other classes within proteobacteria (Additional file 1: Table S1). Conserved domain searches (CD) showed that all the initial protein hits belong to the same broad sequence family as the AT-like and ST-like family sequences (cd08576, GDPD-like SMase D/PLD; E < 1e-10), and not other domain families within the broad GDPD superfamily (see Additional file 1: Table S1). Some hits in lineages such as the Cnidaria or Proteobacteria had such close similarity (E < 1e-25) to ST or AT query sequences as to suggest lateral gene transfer events similar to the one previously identified between fungi and actinobacteria [2]. Meanwhile, a set of 8 relatively weak hits from ST queries, found in aquatic/marine proteobacteria and crustaceans, had high mutual sequence similarity (E < 1e-20 for most pairwise comparisons) and suggested the discovery of a novel, widely distributed, toxin-like sequence family.

To find additional homologs, we performed translated BLAST searches of genome and transcriptome databases, as well as secondary protein and translated BLAST searches from the initial protein BLAST hits above. Secondary searches initiated from the aquatic/marine proteobacterial/crustacean hits led to expansion of this seed group to a wide variety of organisms, almost all from aquatic/marine habitats (Additional file 1: Table S2). Translated genome and transcriptome searches from ST queries led to identification of further close ST homologs in previously unrepresented lineages. Most notably, it solidified the presence of close ST-like homologs in cnidarians and led to the discovery of bona fide close ST-like homologs in myriapods (Additional file 1: Table S3). Meanwhile, translated searches from AT and *Burkholderia* queries, and secondary searches from unclassified query sequences found in the initial blastp search, led to identification of only a few additional homologs (Additional file 1: Tables S4 and S5). These searches set the stage for a new consideration of the evolutionary history of the GDPD-like SMase D/PLD family, including expanded phylogenetic distribution, potential lateral gene transfer events and/or gene loss, and origin from GDPDs.

### Phylogenetic relationships

**Fig. 1:**
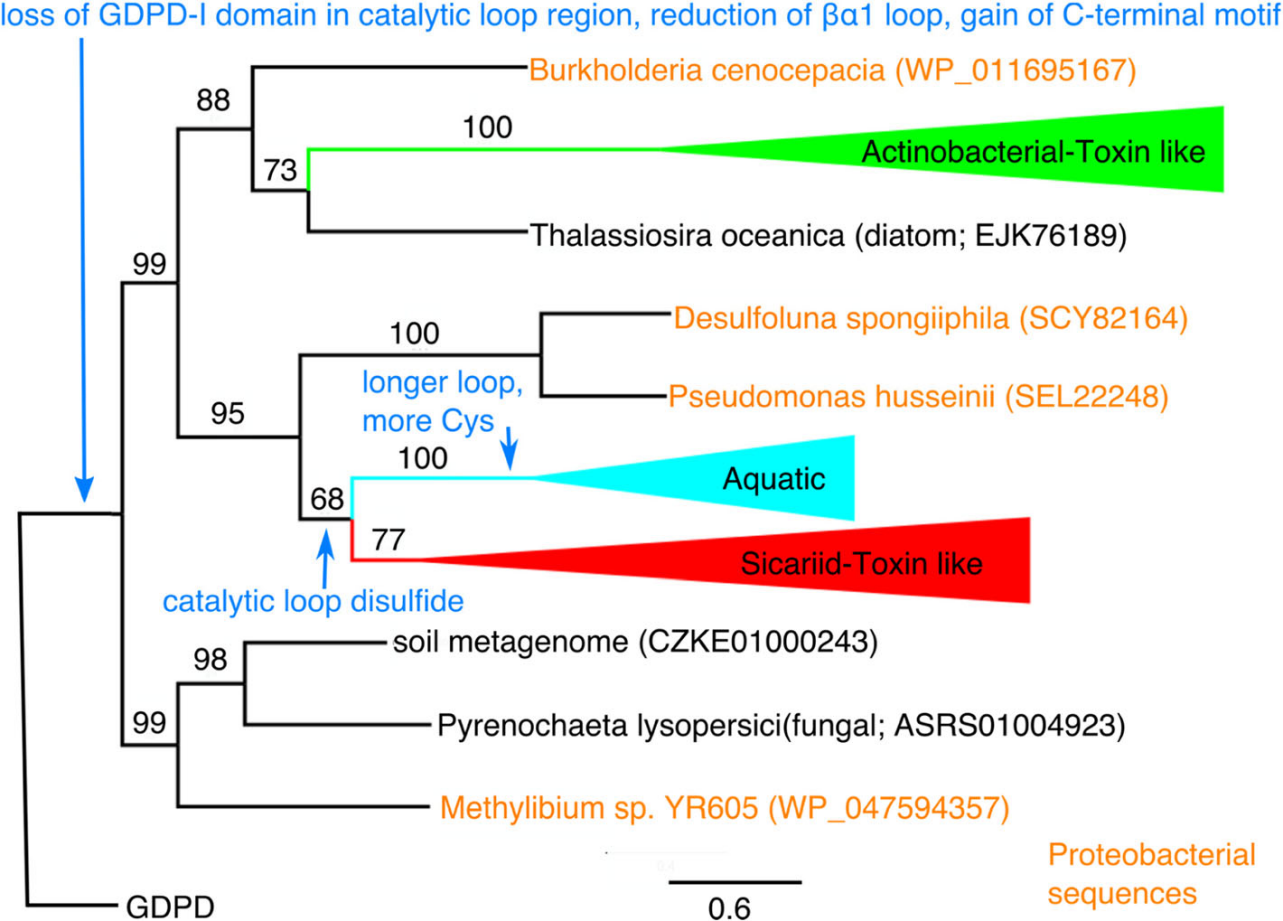
Rooted phylogenetic tree of GDPD-like SMaseD/PLD family. Three major clades are collapsed and colored, with bootstrap values shown on branches. Most basal sequences are of proteobacterial origin (orange). Evolution of signature sequence/structure features is indicated in blue

We aligned the GDPD-like SMase D/PLD sequences (Additional file 2: Figure S1) and constructed a phylogenetic tree using RaxML (Fig. 1). We then rooted the tree using six highly diverse GDPDs of known structure as outgroups, while restricting the character set to positions that could be confidently aligned using structure-structure alignment between GDPDs and PLD toxins (see Materials and Methods). All six outgroups, considered together or separately, rooted the tree on the same branch.

Most sequences belong to one of three major clades, corresponding to expanded versions of the AT-like and ST-like groups, and a third large new group. These clades are collapsed in Fig. 1, but their internal tree structure and taxonomic representation are discussed further below (see also Figs. 5, 6 and 7 and Additional file 1: Tables S2-S7). The expanded AT-like group (Additional file 1: Tables S4 and S7) is recovered as a clade with 100% bootstrap support. The expanded ST-like group (Additional file 1: Tables S3 and S6) is more weakly supported as a clade (77% bootstrap support), but has a clear catalytic loop signature (see below) that distinguishes it from other toxin-like PLDs and supports its monophyly. The third group is a well-supported clade (100% bootstrap support) that includes homologs from diverse organisms found primarily in marine or aquatic habitats. We refer to this novel family as the Aquatic clade (Additional file 1: Table S2). It is sister to the ST-like group, though with weaker bootstrap support (68%).

The tree also includes a scattering of sequences, mostly from proteobacteria, including the singleton sequence from *Burkholderia* found by Felicori and coworkers [2] (Fig. 1; Additional file 1: Table S5). The proteobacterial sequences represent three diverse classes: β (*Burkholderia* and *Methylibium*), γ (*Pseudomonas*) and δ (*Desulfoluna*). Although representation of each class is limited to one or two sequences, proteobacteria resolve as paraphyletic with respect to inclusion of the AT-like clade and the Aquatic/ST-like clade. In particular, the *Methylibium* sequence is sister to all other taxa in the outgroup rooting, while a pair of proteobacterial sequences (γ and δ) is sister to the Aquatic/ST-like clades. The *Burkholderia* singleton is sister to a lineage in which a homolog from the oceanic diatom *Thalassiosira oceanica* is weakly supported as the closest relative of the AT-like group. The paraphyletic grouping of proteobacterial sequences in this analysis suggests divergent origins of the GDPD-like SMaseD/PLD sequences from a proteobacterial ancestor.

### Shared and distinguishing sequence features of the major toxin-like PLD groups

**Fig. 2:**
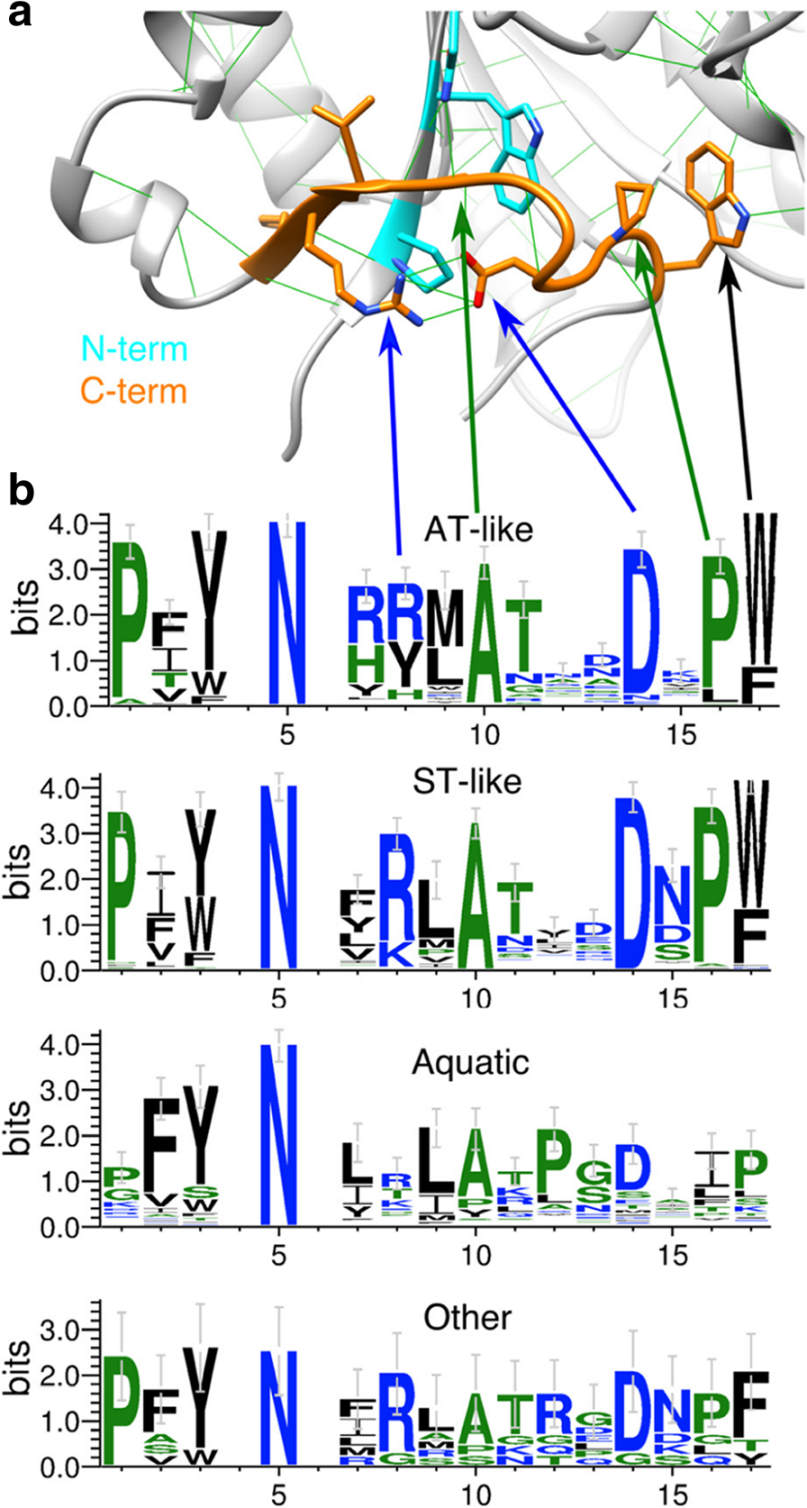
Conservation of stabilizing “plug” motif in GDPD-like SMaseD/PLD. **a** Ribbon diagram showing C-terminal motif (orange) and interacting N-terminal residues (cyan). Most notable in the C-terminal motif are an Arg-Asp (RD) salt bridge (blue arrows), Ala and Pro residues that participate in hydrophobic interactions (green arrows), and a Trp side chain that packs into the bottom of the β-barrel (black arrow). **b** Sequence logos (weblogo.berkeley.edu) depicting residue conservation in the C-terminal motif (positions 7–17 of the logo) plus interacting N-terminal residues (1–3 and 5 of the logo). The actinobacterial-toxin like (AT-like) and sicariid-toxin like (ST-like) clades conserve a very similar motif, as do the basal, proteobacterial dominated sequences (other), while the version of the motif in the Aquatic clade is recognizable but somewhat divergent

One signature feature of the GDPD-like SMaseD/PLDs is a C-terminal “plug” motif (Fig. 2) identified in previous work [1]. Sequence logos (Fig. 2) show that the motif is similar in the ST-like and AT-like groups, as well as in the proteobacterial-dominated basal sequences. The motif profile diverges in the Aquatic group yet retains a similar overall pattern. The C-terminal motif is a synapomorphy reflecting divergence of GDPD-like SmaseD/PLDs from a single ancestor, rather than convergent origin from different GDPDs in diverse lineages.

**Fig. 3:**
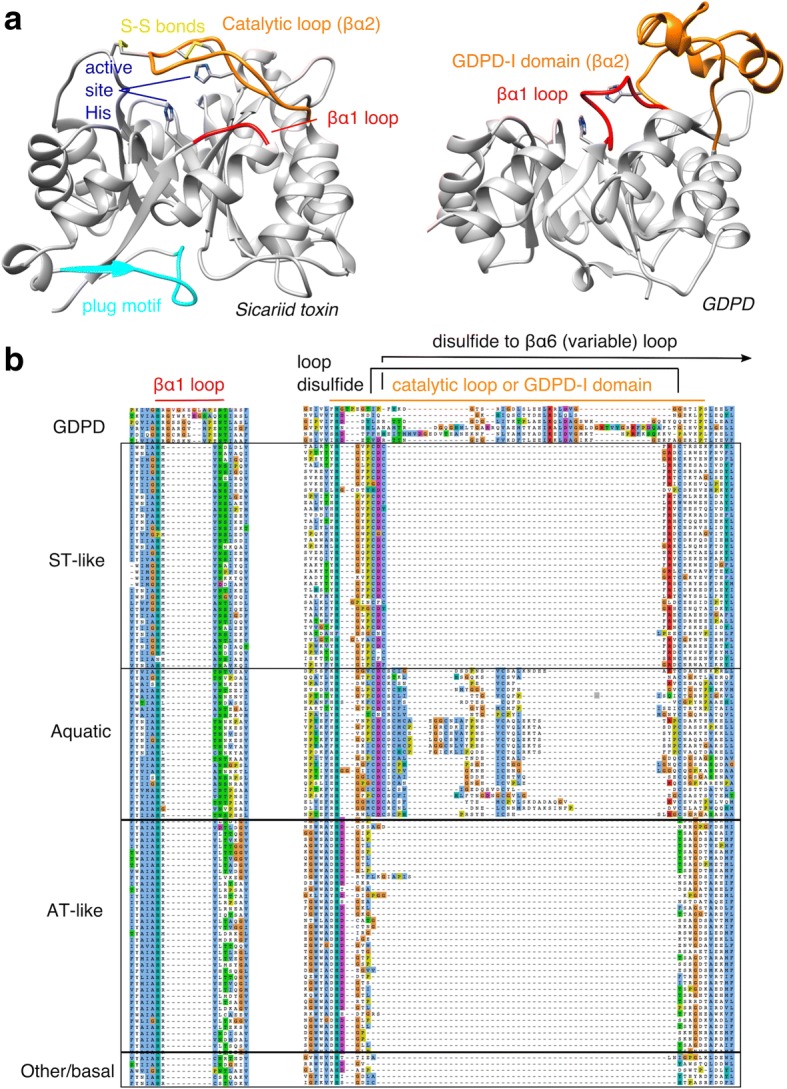
Catalytic and βα1 loops of GDPD-like SMaseD/PLDs. (**a**) Ribbon diagrams of a Sicariid toxin from *Loxosceles intermedia* (PDB ID 3RLH) and a GDPD from *Oleispira antarctica* (PDB ID 3QVQ), with βα1 loop (red) and most of the βα2 region (orange) highlighted, as well as C-terminal plug motif (cyan), disulfide bonds (yellow) active site histidines (blue). (**b**) Partial sequence alignment, including the βα1 loop and catalytic loop (βα2 region), of GDPD-like SMaseD/PLDs filtered at 80% ID. All GDPD-like SMaseD/PLDs have a conserved βα1 loop length that is shorter than the βα1 loop of GDPDs. Catalytic loop of ST-like, AT-like and basal groups is much shorter than the corresponding region in GDPDs, which is an entire small domain (GDPD-I). ST-like and Aquatic have a similar pattern of cysteine residues, but the Aquatic active site loop is longer, variable in length, and has additional cysteine residues

A second signature of GDPD-like SMaseD/PLDs is the length of the short βα1 loop connecting strand β1 to helix α1 (Fig. 3). In GDPD families from prokaryotes to mammals, the βα1 loop is at least six residues longer (most typically 7–9 based on an analysis of the NCBI Conserved Domain database) and contains several conserved residues that interact with an adjacent small domain (GDPD-insert domain or GDPD-I domain [25]) nested within the βα2 loop. In GDPD-like SMaseD/PLDs, the shortening of the βα1 loop may relate to the absence of the adjacent GDPD-I domain (see below). The βα1 loop length is a second signature feature, newly described here, that argues against convergent evolution of GDPD-like SMaseD/PLDs.

The GDPD-I domain is largely deleted in GDPD-like SMaseD/PLDs and replaced by a βα2 loop sequence that differs characteristically between the major groups (Fig. 3). In Sicariid toxins, the βα2 loop is known as the catalytic loop [26]. In the ST-like group it has a conserved length (15 residues), a pair of conserved cysteine residues that form a disulfide bond within the loop, and a third less conserved cysteine that covalently links the βα2 loop to the βα6 loop. In the AT-like group, the catalytic loop is shorter (9–13 residues), more variable, and lacks conserved cysteines. The Aquatic group, meanwhile, conserves the cysteines of the ST-like group but within a longer catalytic loop sequence that contains at least one additional pair of cysteine residues. The signature sequence of the catalytic loop distinguishes the three major families of toxin-like PLDs, though it does not distinguish the AT-like group from the scattered basal sequences. Based on our phylogeny, the ancestral catalytic loop of the GDPD-like SMaseD/PLDs was probably very short and lacked conserved cysteine residues.

### Domain architectures

**Fig. 4:**
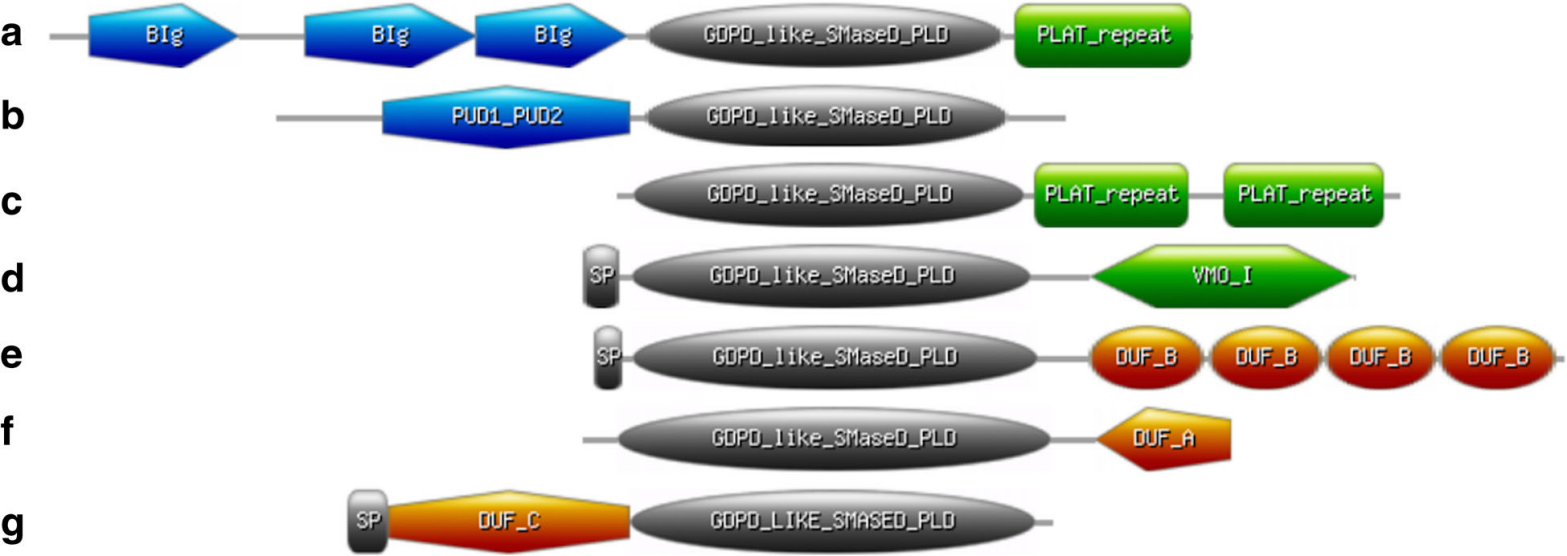
Domain architectures found in GDPD-like SMaseD/PLD homologs, predominantly in basal and Aquatic clade sequences. **a** A basal hypothetical protein sequence from *Pyrenochaeta lysopersici* includes a C-terminal PLAT (Polycystin-1, Lipoxygenase, Alpha-Toxin) repeat domain (cd01756), as well as N-terminal bacterial Ig-like domain repeats (BIg) homologous to those found in calcium-dependent bacterial adhesins (PDB IDs 4P99 and 2YN3), **b** One basal sequence and one AT-like sequence have an N-terminal domain with similarity to PUD-1/PUD-2 from *C. elegans* (protein upregulated in daf-2 loss of function); PDB ID 4JDE), **c** Three basal sequences contain one or two C-terminal PLAT repeats, **d** Several rotifer sequences in the Aquatic clade contain a C-terminal VMO-I domain (vitelline membrane outer layer protein-I; cd00220), **e** At least 70% of Aquatic clade sequences contain 1–4 repeats of an unclassified domain of unknown function (labeled DUF-B) with 10 conserved cysteine residues (Additional file 3: Figure S2), **f** two metagenomic sequences in the Aquatic clade with 74% overall identity conserve a ~ 80-residue Cys-rich domain of unknown function (DUF-A) that is also found in several species of eukaryotic marine phytoplankton, **g** two basal proteobacterial sequences with 46% overall sequence identity conserve an apparent ~ 150-residue N-terminal domain of unknown function (DUF-C). VMO-I and PLAT repeat domains were identified using CD-search on the NCBI Conserved Domain Database, while PUD and BIg domains were identified using FFAS (see Materials and Methods). Signal peptides were not evident on all sequences; in some but not all cases this may be due to an incomplete N terminal sequence

Known sicariid and actinobacterial toxins are single-domain proteins, but all basal sequences and the great majority of Aquatic clade sequences have significant regions of sequence outside of the catalytic domain. Some of these regions contain recognizable domains, yielding novel domain architectures (Fig. 4). Other putative domains resisted classification despite conservation in multiple homologs. At least 70% of Aquatic clade members have one to four repeats of a novel, unclassified cysteine-rich domain (Additional file 3: Figure S2). Three basal sequences have one or more PLAT (Polycystin-1, Lipoxygenase, Alpha-Toxin) domains [27], which commonly exhibit calcium-dependent membrane/lipid recognition and help target proteins to membranes [28–30]. Other domains identified include bacterial Ig-like domain repeats, PUD-1/PUD-2 (protein upregulated in daf-2 loss of function) [31], and VMO-I (vitelline membrane outer layer protein-I) [32] domains. The Ig-like domains are distant homologs of those found in calcium-dependent bacterial adhesins [33, 34], while the biochemical function of PUD-1/PUD-2 is not well understood. VMO-I is probably a carbohydrate-binding domain [35], and it bears mentioning that several AT-like clade homologs also contain domains likely to recognize glycans in surface glycoproteins (Materials and Methods; Additional file 1: Table S7). While the functions of these PLD-fused domains are diverse and in some cases unknown, a role in surface recognition and adhesion is a common theme. The multidomain nature of the basal sequences suggests that the ancestral GDPD-like SMase D/PLD may have utilized additional domains for interfacial and other properties. Finally, a majority of sequences in all three major clades, along with several basal sequences, have recognizable signal peptides (Additional file 1: Tables S2-S7). As expected, most members of this family are probably secreted enzymes.

### Taxonomic distribution of ST-like PLDs

**Fig. 5:**
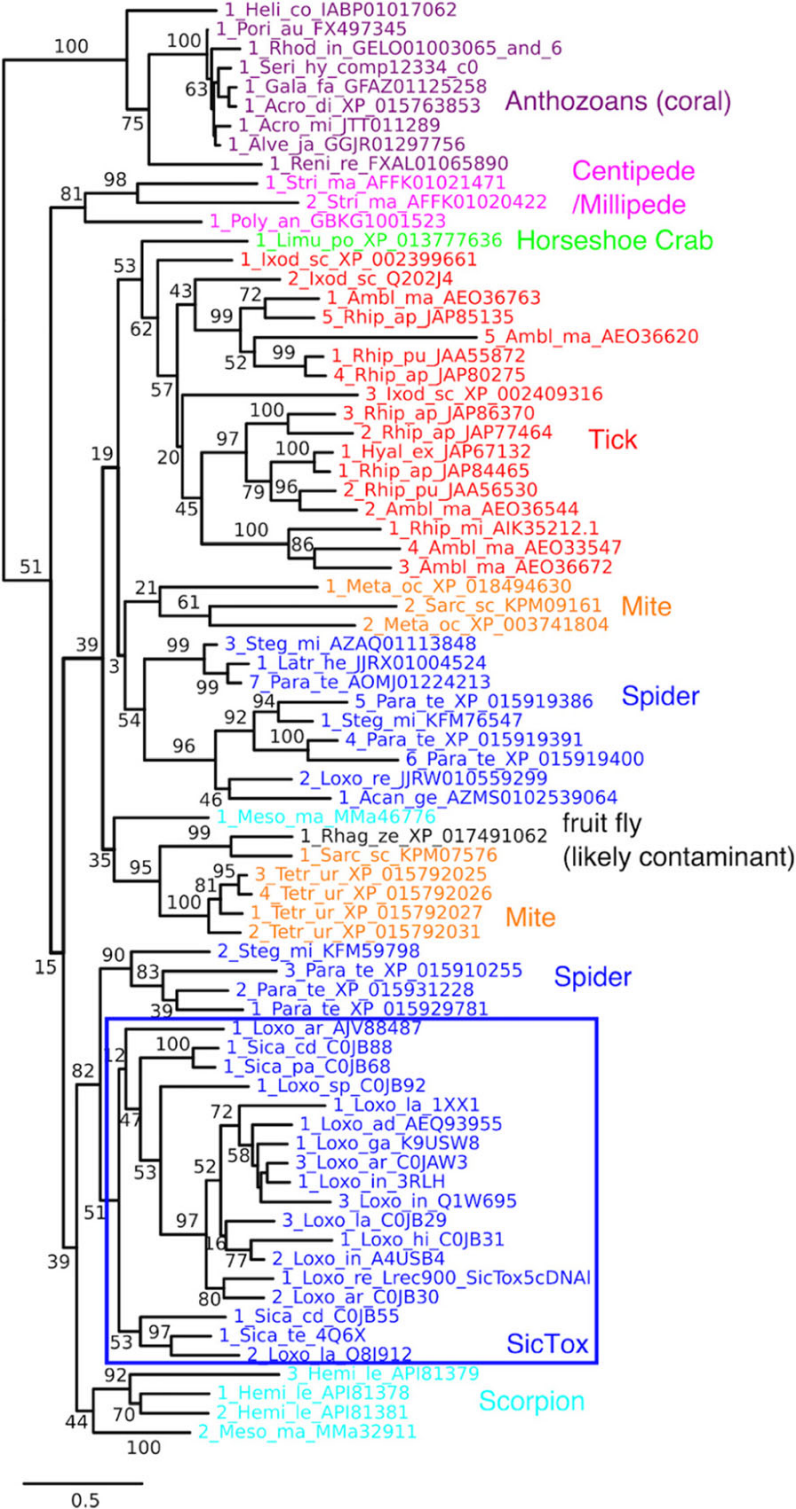
Subtree for Sicariid toxin-like (ST-like) clade, color-coded by organismal classification. The clade corresponding to the Sicariid toxins themselves (SicTox) is highlighted by a blue box. Taxon names include genus and species labels (e.g. Loxo_ar for *Loxosceles arizonica*) as well as specific protein or nucleotide database identifiers

Following reports of sicariid toxin homologs in ticks [8] and mites [2], it seemed possible that this family would prove to be conserved in arachnids and maybe chelicerates in general. Indeed, current BLAST searches with sicariid toxin sequences detect homologs (E-values <1e-20 to Sicariid query sequences) in all 24 available representative chelicerate genomes (see also Fig. 9 below) and many transcriptomes. This includes the horseshoe crab *Limulus polyphemus* [36] and representatives of a wide variety of arachnid orders (including scorpions and acarines) and non-sicariid spiders (Additional file 1: Tables S3 and S6; Fig. 5). Many species carry multiple paralogs, with some copies showing deviations from active site consensus sequences that suggest functional divergence or gene decay.

Outside of chelicerates, we detected arthropod ST-like homologs in a broad set of myriapods (centipedes and millipedes) (Additional file 1: Table S3 and Fig. 5). The genome of the centipede *Strigamia maritima* (class Chilopoda) has two homologs supported by transcript data [37], and the transcriptome of the millipede *Polydesmus angustus* (class Diplopoda) also contains a homolog [38]. Fragmentary BLAST hits also support a presence in the class Symphyla, while the fourth class of myriapods, Pauropoda, is not well represented in databases. Some of these homologs, such as the *P. angustus* sequence, show perfect conservation of canonical sicariid toxin motifs; others, such as the *S. maritima* sequences, show some evidence of divergence or gene degradation. In our maximum likelihood tree, the myriapod sequences are monophyletic and weakly supported as sister to all chelicerate ST-like sequences, suggesting that ST-like sequences could have been present in the common ancestor of myriapods and chelicerates (Fig. 5). Current hypotheses support myriapods as sister to hexapods and crustaceans, forming the group mandibulata, which is then sister to chelicerates [39] (see also Fig. 9, below). Assuming these relationships are correct, and their presence in arthropods represents a single evolutionary origin, ST-like homologs have an ancient origin in an aquatic ancestor of arthropods (> 550 Ma) [40]. If true, the apparent absence of ST-like homologs in crustaceans and hexapods is consistent with a loss of representatives of this molecular clade, before the most recent common ancestor of crustaceans and hexapods (pancrustacea).

Beyond arthropods, the only other metazoan taxon with strong evidence for sicariid toxin-like enzymes is the anthozoan class of Cnidaria, a branch of animals quite distant from arthropods. Remarkably, we detect homologs from four different anthozoan orders (Fig. 5; Additional file 1: Table S3), suggesting an ancient presence in this group. The genomes of the stony coral *Acropora digitifera* (order Scleractinia) [41] and the sea pansy *Renilla reniformis* (order Pennatulacea) [42] both contain genes encoding close homologs (E-value <1e-40 to sicariid toxin query sequences), well supported by transcript data in the case of *Acropora*. Translated BLAST searches using sicariid toxins yielded very similar hits in transcriptome data from eight other anthozoan species, including representatives of two additional orders (Ceriantharia and Corallimorpharia). Despite this extensive representation, BLAST searches using these sequences as queries failed to find ST-like family sequences in four other representative anthozoan genomes, including those of the sea anemones *Nematostella vectensis* and *Exaiptasia pallida* (order Actiniaria) and the stony corals *Orbicella faveolata and Stylophora pistillata* (order Scleractinia). Other anthozoan transcriptomes also lacked hits, except for three sequences in a reference transcriptome assembly of mesenteries/nematosomes/tentacles from *Nematostella* [43] that were unsupported by other transcriptome or genome data for that organism (Additional file 1: Table S3)*.* The deep presence in anthozoans and arthropods, and absence in other metazoan lineages, may be best explained by ancient lateral gene transfer. We return to this point below.

With our expanded understanding of the distribution of, and relationships among, GDPD-like SMaseD/PLDs in the ST-like clade, we confirm broad genomic presence of homologs in chelicerate and myriapod arthopods. Given this distribution, expression in venoms of sicariid spiders represents recruitment from this gene family for venom function [5]. Chelicerate homologs have also convergently emerged for venomous function in ticks [8], and scorpions [9]. The function of ST-like clade members in the other arthropods, and in anthozoans, is unknown.

### Taxonomic distribution of the aquatic clade

**Fig. 6:**
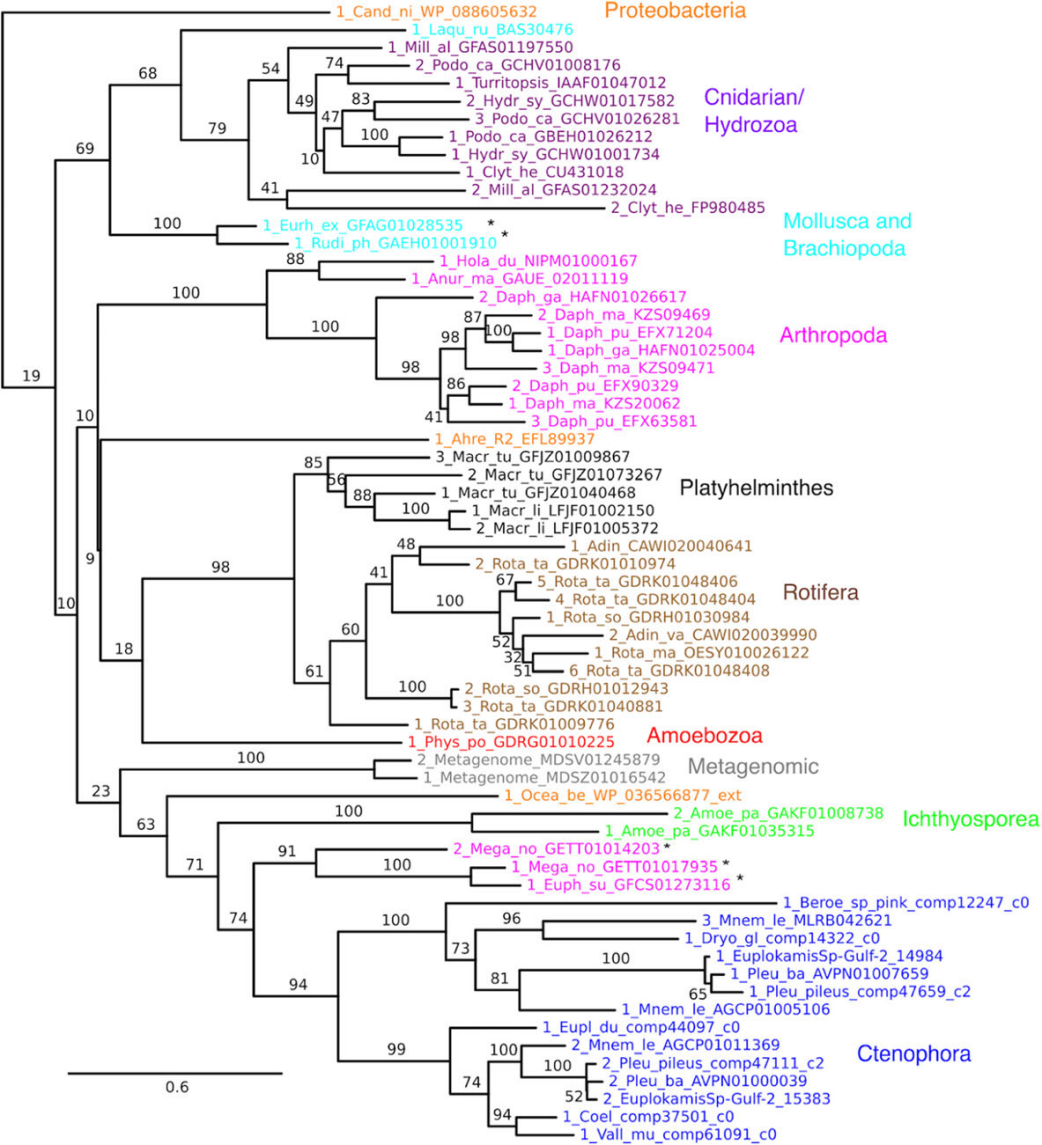
Subtree for Aquatic clade, color-coded by organismal classification. Asterisks indicate lower confidence sequences from organisms with transcriptome-only support, when these occur within sparsely or weakly represented phyla. These include *Euphausia superba* and *Meganyctiphanes norvegica* as representatives of the class Malacostraca, respectively, within the sparsely represented phylum Arthropoda; as well as the two representatives from the phylum *Mollusca*. Taxon names include genus and species labels (e.g. Rota_ta for *Rotaria tardigrada*) as well as specific protein or nucleotide database identifiers

The newly discovered Aquatic clade has a strikingly wide species distribution (Fig. 6; Additional file 1: Table S2). Combined genome and transcriptome data strongly support presence in one prokaryotic phylum and seven eukaryotic phyla: Proteobacteria, Amoebozoa, Ichthyosporea, Ctenophora, Cnidaria, Rotifera, Platyhelminthes, and Arthropoda. Transcriptome and proteome data support a presence in Mollusca/Brachiopoda. Ctenophores are particularly well represented, with homologs found in both representative genomes, *Mnemiopsis leidyi* and *Pleurobrachia bachei*, and in a wide array of transcriptomes from the major ctenophore classes. Thus, the presence of Aquatic clade PLD genes in ctenophores is probably ancient. With the exception of the slime mold *Physarum polycephalum* and the wood-decomposing springtail *Holacanthella duospinosa*, all Aquatic clade members come from organisms that occupy aquatic, marine, or tidal habitats.

Despite their wide species distribution, Aquatic clade homologs have a sparse presence, or no detectable presence, in most metazoan phyla. Within Arthropoda, for example, genome and transcriptome data from multiple species strongly support establishment in the crustacean genus *Daphnia.* The only other unambiguous arthropod representation is in the hexapod order Collembola, supported by genome and transcriptome data in *Holacanthella duospinosa* [44] and by transcriptome data in *Anurida maritima*, both in the family Neanuridae*.* Other crustacean and collembolan genomes lacked detectable homologs, as did all other hexapod genomes including insects (see also Fig. 9, below). However, there is some transcriptomic evidence for a limited presence in the crustacean class Malacostraca. Within Rotifera, homologs are well supported by genome and transcriptome data in the bdelloid rotifers *Adineta vaga* and *Rotaria magnacalcarata* but not found in the genomes of *Adineta ricciae* or *Rotaria macrura* [45], or the non-bdelloid rotifer *Brachionus calyciflorus*. Within Cnidaria, there are Aquatic homologs in at least five genera in the hydrozoan class, including raw genome data from *Hydractinia*, yet no detectable homolog in the genome of the representative hydrozoan *Hydra vulgaris* [46]. Non-metazoan taxa are represented only by one species of slime molds Amoebozoa (*Physarum polycephalum*), one species of the Ichthyosporea (*Amoebidium parasiticum)*, and three diverse species of Proteobacteria (*Ahrensia*, *Oceanospirillum* and *Candidimonas*)*.* Glaring absences include plants, all deuterostomes, nematodes, annelids, most molluscs, insects, and all other bacteria.

A paucity of genome and/or transcriptome data is unlikely to account for sparse or absent representation. Scaffold-level genome assemblies with strong sequence coverage have been deposited in NCBI databases for numerous representatives of each of the above phyla (refer to Fig. 9 below for numbers of genomes as of May 2018). In some individual species, absence of a detectable homolog in a genome could reflect gaps in draft genome assembly. It also cannot be ruled out that acquisition of many introns, or extreme sequence divergence, contributes to apparent absence in some lineages. However, such concerns are mitigated by extensive secondary BLAST searches and other arguments (see further discussion below). On the whole, incomplete or sparse representation in a lineage likely reflects gene loss in some cases and lateral gene transfer in others.

For taxa with multiple Aquatic homologs from different species we were able to test whether the proteins were monophyletic within the taxonomic group (Fig. 6). The broadly represented ctenophore sequences are a clade, within which there is evidence of an ancient duplication leading to distinct paralogs. Hydrozoan homologs and most arthropods (with the exception of the Malacostraca) are each recovered as monophyletic. Support for a flatworm and rotiferan clade is strong (98% bootstrap support), which may reflect their taxonomic relatedness, since both phyla are in the platyzoan clade of spiralia [47, 48]. The patterns of conservation of Aquatic homologs across deeper clades, the ctenophores in particular, suggest that the Aquatic group, like the ST-like group, has an ancient origin.

While support for the Aquatic clade is strong (100% bootstrap), the deeper relationships among the taxon-specific clades are not well resolved. However, tree topology tests allow rejection, at a 95% confidence level, of monophyly for the combined set of protostomian sequences (Additional file 1: Table S8). Arthropod and spiralian (Lophotrochozoa) monophyly are also rejected, with the caveat that sequences from Malacostraca and Mollusca are supported only by transcriptome data, albeit from at least three species each (Additional file 1: Table S2). The lack of congruence between the organismal and molecular phylogeny suggests that the Aquatic clade has experienced either lateral gene transfer or a combination of ancient gene duplication and extensive gene loss, or both (see further discussion below).

### Taxonomic distribution of AT-like group

**Fig. 7:**
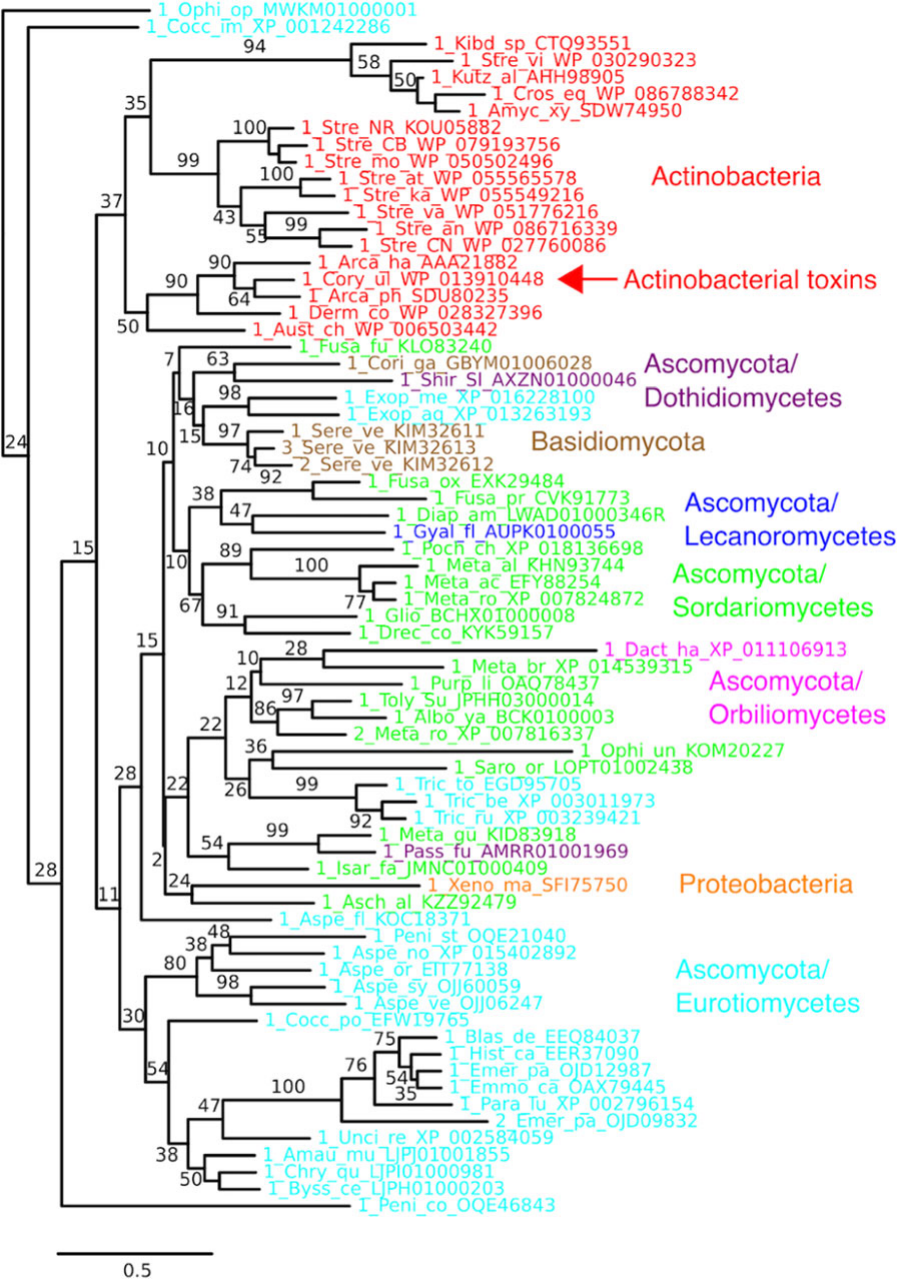
Subtree for Actinobacterial toxin-like (AT-like) clade, color-coded by organismal classification Taxon names include genus and species labels (e.g. Aspe_fl for *Aspergillus flavus*) as well as specific protein or nucleotide database identifiers

Expanding on previous observations, we detected a broad set of fungal and actinobacterial homologs that are strongly supported as a single clade that is not a near relative of ST-like PLDs (Figs. 1 and 7) [2]. The actinobacterial representation includes five orders, adding one order (four genera of Pseudonocardiales) to the taxa detected previously, as well as diversity within the other four orders. Within Ascomycota, we detect representatives from four classes, seven orders, 11 families, and 21 genera, representing an expansion of fungal sequences available in databases since 2013. As with the phylogeny of the Aquatic clade, deeper nodes in this lineage are not well supported. With that caveat, our phylogeny does not support monophyly of Ascomycota, largely because of the inclusion of actinobacteria, one proteobacterium, and the few representative basidiomycota rendering the lineage paraphyletic. Actinobacteria are resolved as a clade, albeit with negligible support (37% bootstrap support).

## Discussion

### Models

Having established that GDPD-like SMaseD/PLDs are a monophyletic group descended from GDPD enzymes, and having reexamined their phylogenetic distribution, we now consider two models to explain the origin of this distribution. One limiting model is that it derives purely from vertical descent accompanied by gene duplication and loss; a second model is that lateral gene transfer also contributed significantly. At first glance, the phylogeny and taxonomic representation of GDPD-like SMaseD/PLDs seems to favor involvement of lateral gene transfer. Evidence includes phylogenetically disparate presence of homologs within and among the three major clades, combined with the absence of homologs in major areas of the tree of life that have representative genomes and transcriptomes (including a majority of bacterial clades, archaea, plants, basal Eukaryotes (Excavata), deuterostomes and hexapods).

**Fig. 8:**
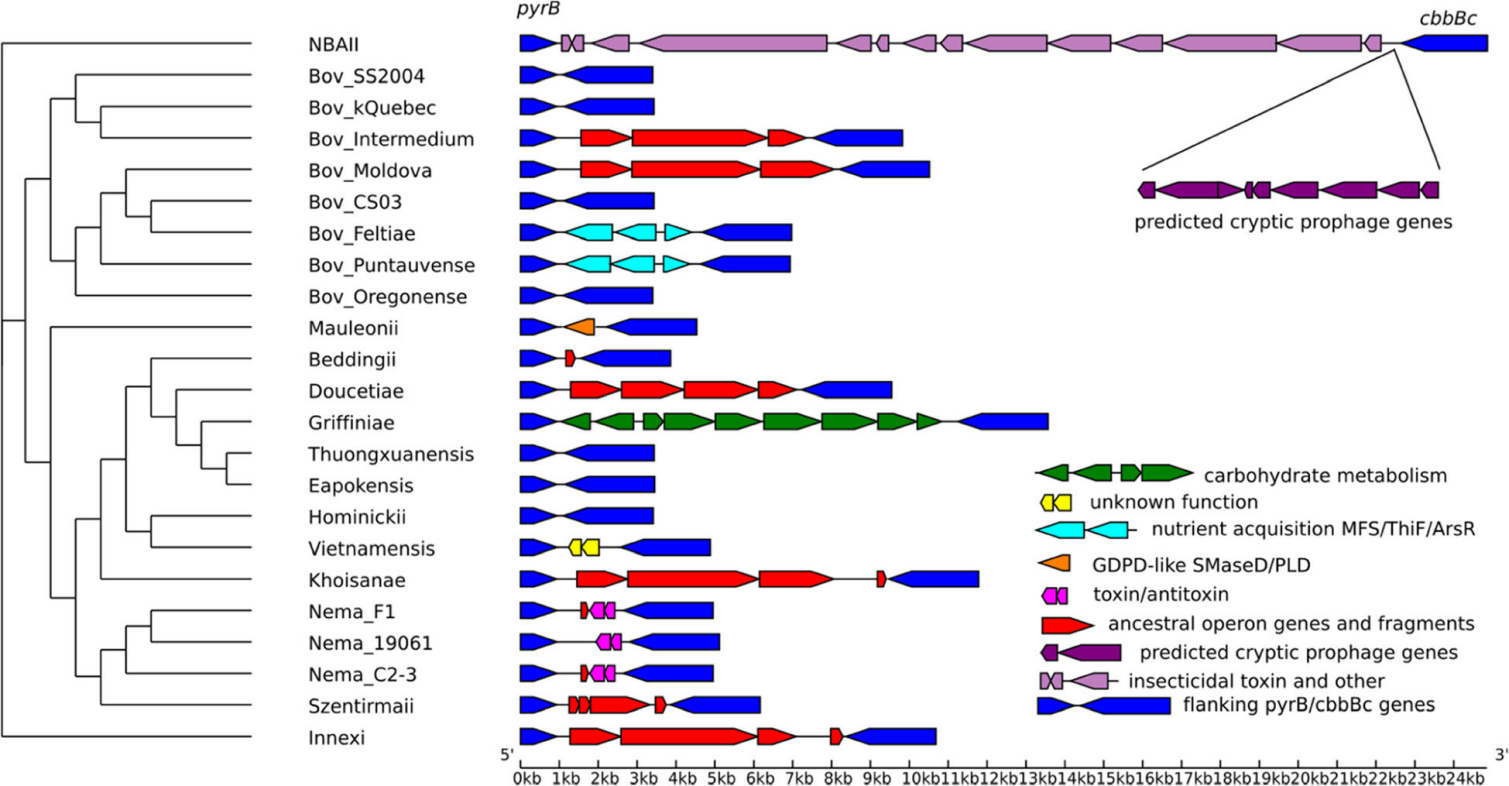
Portion of pyrBI operon region in 23 *Xenorhabdus* species, showing highly diverse gene configurations in the region between *pyrB* and *cbbB*_*c*_. In *X. mauleonii*, this region carries a GDPD-like SMaseD/PLD (AT-like) acquired by lateral gene transfer

Indeed, a pure vertical descent model can be falsified by two clearcut cases of lateral gene transfer within the AT-like group, both involving bacteria. One previously described event, also evident in our analysis, occurred between fungi and actinobacteria, the two major groups of organisms carrying AT-like group PLDs [2]. A second involves *Xenorhabdus mauleonii*, the lone representative of Proteobacteria in the AT-like group (Fig. 6). Dozens of *Xenorhabdus* genomes have been sequenced (Fig. 8), and phylogenetic relationships among them are well characterized [49], but only *X. mauleonii* [50, 51] carries a PLD toxin gene homolog. The homolog in *X. mauleonii* is found within the pyrBI operon [52] and is directly flanked by *pyrB* and *cbbB*_*c*_ [53] (Fig. 8). In other *Xenorhabdus* species, there is either no gene or one of a highly diverse set of modules between *pyrB* and *cbbB*_*c*_, suggesting that this locus is a hotspot for recombination and insertion/deletion. These modules include a toxin-antitoxin pair and prophage genes, genetic elements commonly associated with LGT [54]. The basal species *X. innexi* and a scattering other species contain a gene set that may represent the ancestral gene module in this region. The cross-genome comparisons among *Xenorhabdus* species strongly support the hypothesis that *X. mauleonii* acquired its PLD toxin homolog by LGT. Clearly, it is essential to allow for at least some LGT involving bacteria in the history of the GDPD-like SMaseD/PLDs.

As a side note, the acquisition of a toxin-like PLD by *Xenorhabdus mauleonii* also suggests a functional role in entomopathogenicity. *Xenorhabdus* bacteria are entomopathogenic endosymbionts of entomopathogenic nematodes [55]. Numerous fungi that carry AT-like PLDs are also entomopathogens, including *Aschersonia aleyrodis*, a whitefly control agent [56] with a toxin-like PLD that is sister to *X. mauleonii* in the tree of the AT-like group (Fig. 7). Whether or not the direct source of LGT to *X. mauleonii* was an entomopathogenic fungus, the relatively close homology between PLDs from unrelated entomopathogens suggests that the PLD may somehow foster pathogenicity toward insects.

**Fig. 9:**
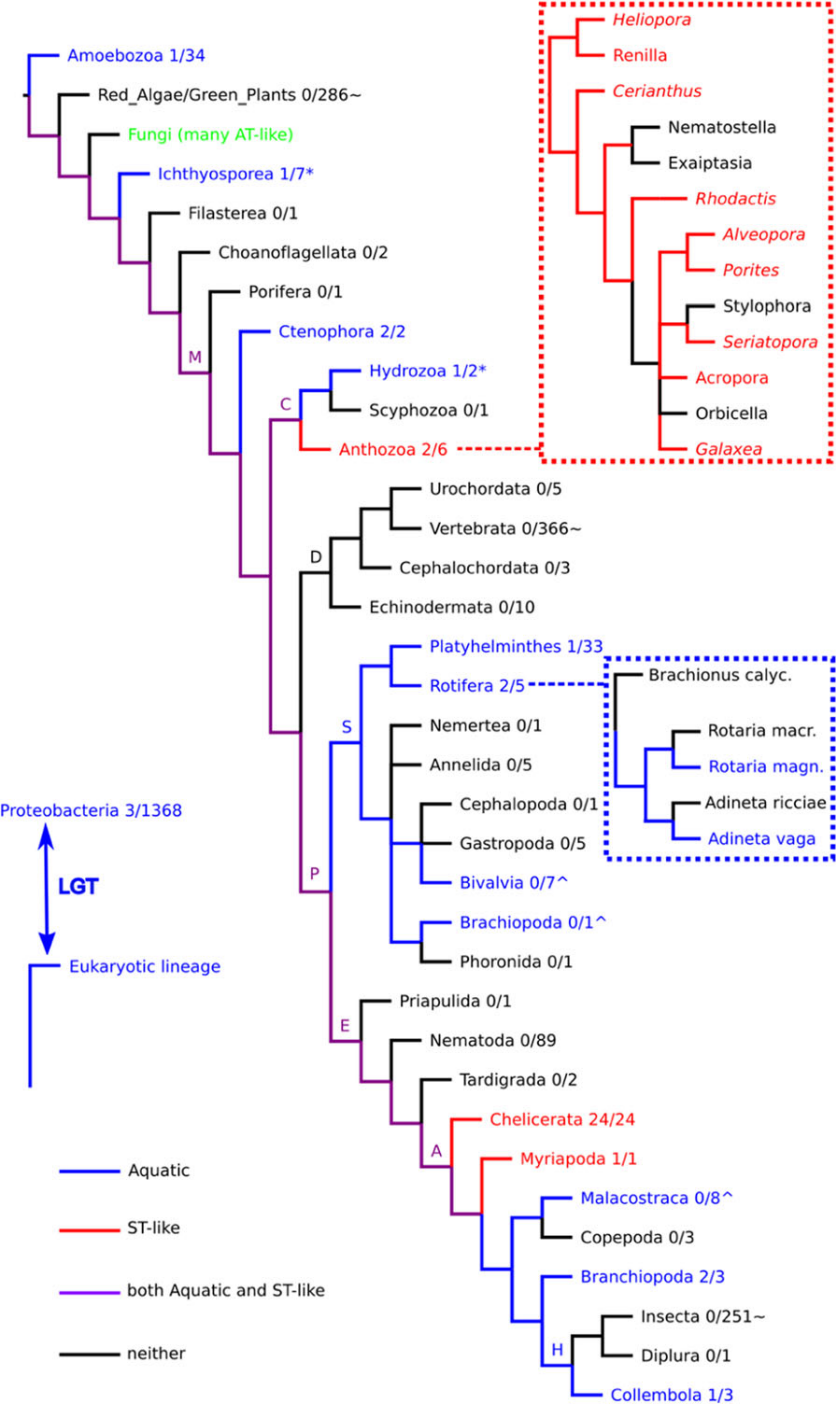
Partial eukaryotic organism tree showing widespread losses of ST-like and/or Aquatic GDPD-like SMaseD/PLD genes, according to a model of ancient duplication followed by vertical descent. Colors indicate nodes and branches retaining both paralogs (purple), ST-like only (red), Aquatic only (blue), or neither (black). Branch labels indicate important clades: M, Metazoa; C, Cnidaria; D, Deuterostomia; P, Protostomia; S, Spiralia; E, Ecdysozoa; A, Arthropoda; H, Hexapoda (see Materials and Methods). Each taxon (phylum or class in most cases) is annotated with the number of genomes containing a PLD gene, divided by the total number of NCBI representative genomes assembled at scaffold level or higher (May 2018). Taxa marked with * also include additional genomes in which PLD genes were detected in unassembled (or assembled but not NCBI-deposited) genomic data. Taxa marked with ^ showed no PLD genes in NCBI representative genomes but did have hits in transcriptomes from multiple genera. Taxa marked with ~ showed one or several hits but with contamination suspected. This tree topology and evolutionary model imply 15 losses of ST-like and 18 losses of Aquatic genes, and additional losses would be necessary to explain incomplete conservation within certain taxa. While such extensive loss seems unlikely, the insets (dashed boxes) show distributions within Anthozoa and Rotifera that are consistent with at least three ST-like or Aquatic gene loss events in these phyla alone. The Anthozoan tree includes genera with transcriptome data only (italicized). The presence of scattered proteobacterial homologs in the Aquatic clade supports a role for lateral gene transfer in contributing to the observed sparse species distribution

We next consider whether vertical descent could nonetheless account for the unusual distribution of GDPD-like SMaseD/PLDs among complex eukaryotes in the ST-like and Aquatic clades (Fig. 9). Both the ST-like and Aquatic clades, which are sister taxa in our phylogeny (Fig. 1), contain representatives of arthropods and cnidarians, albeit from nonoverlapping subgroups. The Aquatic clade, but not the ST-like clade, also contains representatives of many other metazoan and some non-metazoan phyla, while lacking representatives of large metazoan groups like the deuterostomes or insects. Under a vertical descent model, the common ancestor of the ST-like and Aquatic clades must have arisen in some pre-metazoan eukaryote, then undergone at least one gene duplication to give the ST-like and Aquatic clades, followed by variable paralog retention within both cnidarians and arthropods. A number of major cnidarians (e.g. *Hydra vulgaris* and *Exaiptasia pallida*), arthropods (e.g. essentially all hexapods), deuterostomes, and numerous entire metazoan phyla must have lost both paralogs, being unrepresented in either clade. A vertical descent model would thus require massive gene loss in both the ST-like and Aquatic clades (Fig. 9).

Extreme sequence divergence, leading to undetected homologs, could exaggerate the appearance of gene loss, but we doubt this is an issue in our study. In exhaustive PSI-BLAST searches restricted to metazoans, the closest additional hits we found had E-values above 0.1 and belonged largely to the GDE4 family. GDE4-like proteins, which are widespread in metazoans, have signature GDPD features and are therefore not a highly diverged branch of the GDPD-like SMaseD/PLDs, though they do have lysophospholipase D activity. PSI-BLAST searches not restricted to metazoans were quickly swamped by bacterial hits belonging to GDPD families. Thus, any undetected, highly diverged sequences from hexapods or deuterostomes (metazoans that are well represented in protein databases) would have to be more different from GDPD-like SMaseD/PLDs than GDPD-like SMaseD/PLDs are from major groups of GDPD enzymes.

We also noted above that the molecular phylogeny of the Aquatic clade is not fully congruent with deep organismal relationships among eukaryotes (Additional file 1: Table S8). Under a vertical descent model, ancient duplication events within the Aquatic clade, along with even more paralog loss, would have to be invoked to resolve this conflict. When one further considers the scattering of proteobacterial sequences in the Aquatic clade, LGT becomes a more sensible explanation. That having been said, patterns of conservation with certain lineages, such as Anthozoa and Rotifera, do point toward multiple recent gene loss events (Fig. 9, dashed boxes), and extensive gene duplication is also evident in both the ST-like and Aquatic clades (Figs. 5 and 6). While gene duplication and loss by themselves are unlikely to fully explain the observed phylogenetic distribution in these clades, they should not be minimized as contributors.

We suggest that an ancient LGT-mediated radiation, between and among proteobacteria and various eukaryotes, contributed significantly to the observed phylogenetic distribution, in combination with extensive gene duplication/loss. As noted above, the presence of mostly proteobacterial homologs among basal sequences suggests a bacterial ancestor for GDPD-like SMaseD/PLDs. These deep homologs are broadly dispersed among the β, γ and δ proteobacteria, while a few additional homologs from γ and α proteobacteria are also scattered within the major clades. Several of the proteobacterial homologs are found on mobile genetic elements, consistent with the propensity for LGT, particularly of secreted pathogenic molecules [57]. The previously identified *Burkholderia cenocepacia* sequence [2], for example, resides on a conjugative or mobilizable plasmid that carries an extensive set of DNA transfer genes, including a conjugative relaxase [58]. The basal sequence from *Methylibium* resides on a predicted genomic island, as does a sequence from *Ahrensia* within the Aquatic clade (see Materials and Methods). The sparse presence of GDPD-like SMaseD/PLDs in the proteobacteria, which are widely represented in genome sequencing projects, thus almost certainly results from their high genetic mobility. Given that interkingdom transfer is well supported within the AT-like clade, it is reasonable to suggest that early GDPD-like SMaseD/PLDs radiated widely through LGT, not only among proteobacteria but also between proteobacteria and eukaryotes.

### Putative functions of aquatic clade proteins

The AT-like and ST-like groups contain homologs that are known toxins, though some homologs could have other functions. Meanwhile, the newly discovered Aquatic group has not been established to contain toxins, and several lines of evidence suggest a diverse array of functions. For example, the slime mold *Physarum polycephalum*, which expresses a member of the Aquatic clade, produces a biologically active cyclic lysophosphatidic acid (cPA) that inhibits cell proliferation [59–61]. Given that Sicariid PLD toxins generate cPA from lysophospholipid substrates [24], the Aquatic clade homolog is a candidate for the cPA synthase in *Physarum*. It is noteworthy that the cPA was isolated from a single-celled form of the organism, the myxamoebae, and the strongest expression evidence for the Aquatic PLD also comes from single-cell transcriptomes. Two other members of the Aquatic clade, from the brachiopod *Laqueus rubellus* and the hydrozoan *Hydractinia symbiolongicarpus*, show possible evidence for tissue-specific expression. The *Hydractinia* sequence was identified from a study of transcriptomes in feeding, reproductive and defensive polyps, and was expressed specifically in feeding polyps [62]. The *Laqueus* sequence was reported in a proteomic and transcriptomic study of the brachiopod shell matrix, and was identified specifically from the insoluble organic matrix [63]. These observations suggest that members of the Aquatic clade may play diverse biological roles. The majority of members of this family also have one or more copies of a ~ 70-residue cysteine-rich C-terminal domain (Additional file 3: Figure S2). This domain may ultimately provide clues to Aquatic clade function, but as of now it has no apparent homology to any known domain family.

## Conclusions

The GDPD-like SMaseD/PLD enzymes share a single common ancestor and are a monophyletic domain family. The ancestor evolved from a GDPD enzyme, most likely a multidomain protein from bacteria, and acquired several novel features including shortened βα1 and βα2 loops and a C-terminal motif. Descendants of this ancestor have radiated extensively, at least in part by ancient lateral gene transfer. Three major clades emerged, with one (ST-like) now found in corals and arthropods, one (AT-like) in bacteria and fungi, and a third (Aquatic) in a wide array of aquatic organisms. GDPD-like SMaseD/PLDs are ancient and broadly established within a few major lineages, such as the chelicerates and ctenophores. Overall, however, the evolution of this family appears highly dynamic and includes gene duplication and gene loss, in addition to extensive lateral gene transfer. Traditionally known as toxins, GDPD-like SMaseD/PLD enzymes may carry out a wide array of biological functions, which may be illuminated by future investigations.

## Materials and Methods

### Initial protein BLAST searches

Initial protein BLAST (blastp) searches of the NCBI nonredundant protein database, using three sicariid toxins with known structure as query sequences (PDB IDs 4Q6X, 3RLH and 1XX1), yielded over 300 high-coverage (> 75%) hits to proteins from 35 organisms in the class Arachnida (all at E-values <1e-25), including hits to 27 Sicariid spiders, 2 non-Sicariid spiders, and 6 mite and tick species (Acari). A single high-coverage hit was also retrieved for a homolog in Merostomata (horseshoe crab, E = 1e-50 to 1e-63, 35–39% ID), a distantly related class within the arthropod subphylum Chelicerata. This suggested broad conservation of SicTox-like sequences within the Chelicerata. Surprisingly, two high-coverage hits at this high level of similarity (E < 1e-32 to all queries) were also found to hypothetical non-chelicerate proteins, one from *Acropora digitifera* (a stony coral, in the phylum Cnidaria) and one from *Rhagoletis zephyria* (snowberry fruit fly, an insect).

The same searches yielded 11 weaker but significant high-coverage hits to non-chelicerate proteins (1e-17 < E < 1e-03, and 24–31% sequence identity, to at least two of the three SicTox query sequences). These included single hypothetical protein sequences from five highly diverse proteobacterial species representing the α-, β-, γ- and δ-proteobacteria (*Ahrensia sp. R2A130, Oceanospirillum beijerinckii DSM 7166, Methylibium sp. YR605*, *Pseudomonas hussainii* and *Desulfoluna spongiphila*), along with six sequences from two water flea (a crustacean) species, *Daphnia pulex* and *Daphnia magna*. Each of these proteins showed similarity to at least 1 chelicerate SicTox homolog at E-value <1e-10 when used as queries themselves, while showing no similarity below E < 1e-05 to the group of bacterial/fungal PLD homologs described in Dias-Lopes et al. [2] A set of eight of these sequences also showed higher similarity to each other (E-values <1e-20) than to the chelicerate SicTox proteins or to the *Acropora* and *Rhagoletis* sequences described above. These findings suggested the existence of a novel family of GDPD-like SMase D/PLDs, reasonably closely related to the ST-like family but distinct from it.

Initial blastp searches of the NCBI nonredundant protein database were also conducted using three AT-like (actinobacterial or ascomycotal) PLD toxin representatives as query sequences. Two query sequences were selected based on recent biochemical characterization [19]: one (Genbank: AAA21882) from the actinobacterium *Arcanobacterium haemolyticum* and one *(*Genbank: EFW19765) from the fungus *Coccidioides posadasii*, a representative of the Eurotiomycete class. A third query sequence (Genbank: EFY88254) was selected from *Metarhizium acridum CQMa102*, a representative of the Sordariomycete class and a basal fungal sequence in the phylogenetic analysis of Dias-Lopes et al. [2]. These searches yielded a combined total of 249 unique high-coverage hits at E < 1e-25. This dataset included representatives of 9 genera, 5 families and 5 orders within the Actinobacteria; and 19 genera, 9 families, 5 orders and 3 classes within Ascomycota. In addition, outside of these lineages, very strong hits (E < 1e-50 to all queries) were found to a hypothetical protein in *Xenorhabdus maleonii*, a proteobacterium, and *Serendipita vermifera*, a fungus in the phylum Basidiomycota. Finally, at a much lower level of similarity, a weak but high-coverage hit was observed from all queries (at E < 1e-03) to a hypothetical protein from the oceanic diatom *Thalassiosira oceanica*. A blastp search with the *Thalassiosira* sequence showed similarity to 8 actinobacterial/fungal PLD homologs at E < 1e-05. Thus, the new searches revealed that the AT-like family has clear homologs (some very close and some relatively distant) in at least three species outside of Actinobacteria and Ascomycota.

Finally, a blastp search was conducted with a singleton sequence from *Burkholderia cenopacia*, reported as a toxin-like PLD by Dias-Lopes et al. [2] This search returned the *Thalassiosira oceanica* sequence mentioned above as a strong hit (E~1e-30).

Protein BLAST hits from newly represented lineages are summarized in Additional file 1: Table S1. Representative hits (see sequence filtering below) from arachnids, actinobacteria and ascomycota are included in Tables S6 and S7.

### Translated blast and other database searching

To supplement the updated protein database for GDPD-like SMase D/PLDs, an exhaustive set of translated BLAST (tblastn) searches was then conducted on NCBI whole genome shotgun (WGS), transcriptome (TSA) and EST databases across all living organisms, along with blastp searches of the NCBI transcriptome protein database. These searches employed the original query sequences (see above) but also included searches initiated from homologs from newly represented lineages identified in the original blastp searches. Hits resulting from these searches are included in Additional file 1: Tables S2-S5. For the novel Aquatic group (Additional file 1: Table S2) and to some extent for the ST-like group (Additional file 1: Table S3), these searches revealed interesting species distributions, and special care was taken to obtain the most complete and accurate phylogenetic representation (see below).

For newly represented phyla, these searches were further supplemented, where possible, with deeper analysis on web databases dedicated to particular organisms or phyla, or on NCBI sequence read archive (SRA) datasets. This included analysis of ctenophore transcriptome data at http://neurobase.rc.ufl.edu/pleurobrachia; *Mnemiopsis leidyi* genome/transcriptome data at http://research.nhgri.nih.gov/mnemiopsis; *Daphnia* genome data at http://wfleabase.org and http://genome.jgi.doe.gov/; *Acropora digitifera* genome data at http://marinegenomics.oist.jp/coral; *Physarum polycephalum* genome data at http://www.physarum-blast.ovgu.de; *Adineta vaga* genome/transcriptome data at http://genoscope.cns.fr/adineta; *Macrostomum lignano* genome/transcriptome data at www.macgenome.org; *Strigamia maritima* genome data at https://www.hgsc.bcm.edu/arthropods/geophilimorph-centipede-genome-project and http://metazoa.ensembl.org; anthozoan transcriptome data at http://people.oregonstate.edu/~meyere/data.html (keyword search); NCBI SRA genome sequencing data for *Amoebidium parasiticum*; and a draft assembly for *Hydractinia echinata* at https://bica.nhgri.nih.gov/hydractinia/. These analyses led, for example, to the inference of widespread representation of the Aquatic group in the phylum Ctenophora, and to genome-level confirmation of representation in the hydrozoan class within Cnidaria.

There was some concern that homologous sequences could be missed in genome searches due to relatively low sequence similarity to the query, combined with interruption by introns. An illustrative example is the member of the Aquatic group from *Physarum polycephalum*. In tblastn searches of the *Physarum* transcriptome, all three of the original Aquatic group queries used gave strong hits (E-value <1e-25) to the homologous transcript; by contrast, none of the queries yielded hits to sequences in the *Physarum* genome. When the translated *Physarum* transcript was used as a query, however, a set of strong hits to the *Physarum* genome emerged. The reason for this discrepancy is that the *Physarum* homolog is relatively distant from the query sequences and its gene has at least 6 introns.

To minimize missed homologs in translated genomes, a second round of tblastn searches was done using representatives of a particular phylum or class against all genomes within that phylum or class. Some of these searches confirmed conspicuous absences suggestive of either gene loss or lateral gene transfer. For example, searches with hydrozoan queries in the Aquatic group gave no hits in the representative hydrozoan genome *Hydra vulgaris*, other than to a cysteine-rich C-terminal domain; searches with anthozoan queries in the ST-like group gave no hits in several anthozoan genomes including *Nematostella vectensis*, *Exaiptasia pallida, Orbicella faveolata*, and *Stylophora pistillata.* Further examples are discussed within the main text.

For transcriptome hits, hypothetical amino-acid sequences were generally inferred by simple open reading frame analysis of the mRNA sequence, using either DNA Strider or NCBI ORF Finder. Some genome hits were supported by transcript data and vice versa, and in such cases gene models were often available for inference of a hypothetical amino-acid sequence. In certain cases, however, existing gene models appeared to be incorrect. For example, one gene model had a PLD domain within the 5’-UTR of a gene, and more detailed analysis of SRA transcript data suggested that an intron had been missed. In such cases, alternative gene models were used to obtain hypothetical amino-acid sequences. Finally, some translated genome hits lacked any associated transcript data or gene model, but belonged to apparently single-exon genes, based on analysis with NCBI ORF Finder and inspection of amino-acid sequence alignments with homologs. In these cases, hypothetical protein sequences were inferred from direct translation of the genome sequence.

### Filtering of sequences

#### Arachnid sequences

BLAST hits to SicTox queries were dominated by arachnid homologs, and within arachnids, sicariid spider homologs dominate. In generating a representative set of arachnid sequences for further analysis, the sicariid hits were discarded and replaced with a representative set of 18 sequences spanning the known phylogeny of SicTox proteins from sicariids, and including the three initial query sequences of known structure (4Q6X, 1XX1 and 3RLH). Sequences from other arachnids were filtered at 95% redundancy but were otherwise retained unless they were highly incomplete at the termini or contained large deletions. One hypothetical protein sequence from *Stegodyphus mimosarum* (KFM59798) was retained for phylogenetic analysis despite containing only 75% of a PLD domain, due to its importance as a representative of close SicTox homologs in non-sicariid spiders. The final representative set of arachnid sequences (Additional file 1: Table S6; all ultimately assigned to the ST-like clade) contained a total of 62 sequences, including 32 representatives from spiders, 17 from ticks, 8 from mites and 5 from scorpions. While most arachnid sequences came directly from the nonredundant NCBI protein database, 13 tick sequences were derived from the transcriptome shotgun assembly protein database, and 5 hypothetical spider protein sequences were derived from translated BLAST hits from genomes, in cases where the entire PLD domain appeared to reside within a single exon. Sequences from the scorpion *Mesobuthus martensii* were derived from translated BLAST hits that were then mapped to protein sequence models downloaded from http://lifecenter.sgst.cn/main/en/scorpion.jsp.

#### Ascomycotal and actinobacterial sequences

BLAST hits to actinobacterial and fungal queries (AT-like family) were dominated by homologs from these two lineages, and a fairly strict approach was taken in choosing a representative set for further analysis. First, highly incomplete sequences (< 225 residues in length), and sequences with large deletions that included conserved active site residues, were removed unless otherwise specified. The remaining sequences were then filtered for redundancy at 90% identity. Two small sequence subfamilies were also removed from the dataset because they are likely to have diverged functionally and may lack PLD activity, however they may be interesting subjects for future investigation. First, one group of fungal sequences (XP_014576785 from *Metarhizium majus*, XP_007808303 from *Metarhizium acridum*, and XP_018143442 from *Pochonia chlamydospora*) exhibited extremely divergent active sites, including nonconservative active site replacements at His 12, Glu 32 and His 47, and in some cases also Asp 34, Asp 91 and Lys 93. Second, a group of bacterial sequences (WP_083462538 from *Kitasatospora griseola* and WP_037599565 from *Streptacidiphilus rugosus*) exhibited a considerably longer active site loop lacking any histidine corresponding to His 47. This group of sequences also contained probable N-terminal carbohydrate- or actin-binding domains, while almost all other AT-like sequences are single-domain proteins. Aside from those sequences, all ascomycotal and actinobacterial genera represented in the original set of BLAST hits were represented in the filtered set, except for *Hirsutella*, a fungal genus with only a fragmentary blastp hit. The representative protein alignment was supplemented with 14 translated, putatively intronless sequences obtained from tblastn searches of whole genome shotgun or transcriptome data. Sequences were only added if they represented new genera, and were also filtered at 90% redundancy. New phyla represented by these sequences included two different additional classes in the Ascomycota (Dothideomycetes and Lecanoromycetes). The final representative set of homologs from Actinobacteria and Ascomycota (Additional file 1: Table S7; all eventually assigned to the same AT-like clade) contained 72 sequences from 9 genera, 5 families and 5 orders within Actinobacteria; and 32 genera, 14 families, 9 orders and 5 classes of Ascomycota.

#### Sequences from other lineages

In general, sequences from newly represented lineages were retained for phylogenetic analysis unless they were highly redundant (95% ID level), highly fragmentary (e.g. < 75% complete PLD domain), contained major deletions, or were strongly suspected of being contaminants (see below).

#### Probable contaminants

Among ST-like proteins, several sequences putatively belonging to plants (*Humulus lupulus* and *Ambrosia trifida*) proved to be identical to ST-like proteins from plant-feeding mites (*Tetranychus urticae*). On the basis of this apparent instance of mite contamination, ST-like sequences outside of chelicerates showing high identity (> 50%) to known mite sequences were flagged as possible contaminants (see Additional file 1: Table S3). These included two hits putatively from the genome of the snowberry fruit fly *Rhagoletis zephyria*, which is in an early state of assembly at present; most hits from plant transcriptomes; and a fragmentary hit from the crustacean *Talitrus saltator.* The *Rhagoletis* hits are on relatively short unplaced scaffolds and could not be verified with available transcript data, nor were they supported by transcript data from *Rhagoletis pomonella*. In the AT-like group, two Blastp hits (JAV87767 and JAV94811) were recovered from fragmentary sequences putatively from *Photinus pyralis*, a species of firefly. These *Photinus* hits were derived from transcriptome shotgun data, and both are > 80% identical to proteins from fungi in the genus *Metarhizium*, which is comprised of entomopathogens. Cross-species contamination is strongly suspected here as well. Within the Aquatic group, several sequences putatively belonging to *Oreochromis niloticus* (a fish commonly known as tilapia) proved to be identical to confirmed genome and transcriptome sequences from *Amoebidium parasiticum*, a microorganism that is not found in association with tilapia but which had been sequenced at the same institute (Broad Institute). The sequences above were generally removed for phylogenetic analysis, except to illustrate contamination in the case of a *Rhagoletis* sequence (Fig. 5).

#### Low-confidence sequences

Sporadic hits from weakly represented lineages may be regarded as suspect, or tentative, even if no likely source of contamination can be identified. Specifically, some hits assigned to the Aquatic group (see Additional file 1: Table S2) came from transcriptome or genome data in phyla/classes for which a presence was not supported by at least two types of data (e.g. genome/transcriptome or transcriptome/proteome). These included the crustacean subphylum/class Crustacea/Malacostraca within the phylum Arthropoda (transcriptome hits from three species); the class Bivalvia within the phylum Mollusca (transcriptome hits from four species); the classes Alphaproteobacteria, Betaproteobacteria and Gammaproteobacteria within the bacterial phylum Proteobacteria (1 protein hit each, inferred from genomic data). In the case of the proteobacterial hits, contamination is unlikely because of the presence of genes on the DNA contig/scaffold with close homologs in the genome of species in the same genus. In the other cases, the sequences were retained for phylogenetic analyses but were flagged as lower confidence representatives in Additional file 1: Table S2, and in Fig. 6.

### Sequence alignment and phylogenetic tree construction

Sequences were aligned using ClustalX [64]. Maximum-likelihood (ML) phylogenetic trees were constructed with RaxML [65] at the CIPRES Science Gateway (https://www.phylo.org), using WAG + γ4 models with observed frequencies, as recommended by analysis using ProtTest [66]. Although many homologs contain additional domains, especially at the C terminus, only the catalytic PLD domain sequence was used for tree construction, not including N-terminal signal sequences. Trees were rooted in RaxML using 6 GDPD sequences of known structure as outgroups. GDPD of known structure were judged to be best for outgroup rooting, as they allow for the highest possible quality sequence alignment to the ingroup using structure-structure alignment. Structural similarity searches were conducted using VAST [67] with known SicTox structures as query structures, and the four most similar GDPD structures were chosen (3QVQ, 3NO3, 3 L12 an 2O55). Second, two-round PSI-BLAST searches of the PDB were conducted with Sicariid toxins of known structure as queries, and the three best GDPD hits were chosen (3 L12, 2PZ0 and 2CH0). This produced a total of 6 candidate GDPD outgroups from the bacterial phyla Proteobacteria, Bacteroidetes and Firmicutes, as well as one sequence from the red algae *Galdieria sulphuraria*. We aligned the structures and sequences using PROMALS3D [68] and Chimera. For rooting, the character set was limited to 128 best-aligned positions, including the β-barrel framework plus helices α1, α2, and parts of α3, α7 and α8. These regions corresponded to sequence blocks that were 1) alignable within 5 Å in a Chimera Matchmaker alignment, and 2) only the blocks within that set where the Chimera and PROMALS3D alignments agreed. During rooting no restrictions were placed on the ingroup topology. All 6 outgroups, individually and together, rooted the tree on the same branch in the best ML tree. Although the structural alignment introduces a potential bias toward rooting in the Sicariid toxin-like group, the root position lies outside of it.

Phylogenetic hypothesis testing for the Aquatic clade was performed by calculating best maximum likelihood trees with and without a multifurcating constraint tree representing each hypothetical monophyletic group. Statistical tree topology tests (approximately unbiased test) [69] were then conducted as implemented on the IQ-Tree Web Server (http://iqtree.cibiv.univie.ac.at/) [70]. Tests were performed for various eukaryotic clade hypotheses on datasets both including and excluding bacterial sequences.

A partial phylogenetic tree for eukaryotic organisms (Fig. 9) was constructed based on a variety of literature sources. Ecdysozoan phylogeny was based on the following references: [39, 71, 72]. Spiralian (Lophotrochozoa) phylogeny was based on the following references: [48, 73]. Cnidarian phylogeny was based on the following references: [42, 74].

### Analysis of domains, operons and genomic islands

Domain families were identified using batch CD-search at the NCBI Conserved Domain Database, with E-values of 1e-05 or less being accepted as significant hits. Signal peptides were identified using SignalP version 4.0 [75]. Sequences that included substantial N- or C-terminal regions outside the GDPD-like SMaseD/PLD region were also analyzed using FFAS03 against the Pfam and PDB databases, in an effort to identify more remote homologies missed by CD-search [76]. FFAS scores lower than − 10 were considered significant. Proteobacterial genomic DNA was analyzed for genomic islands with Island Viewer 3 (http://www.pathogenomics.sfu.ca/islandviewer3/browse/), which integrates the programs SIGI-HMM, Island Pick and IslandPath-DIMOB [77]. Prophage regions were predicted using PHAST [78]. The genes of the plasmid from *Burkholderia cenocepacia* strain HI2424 were analyzed by BLAST and found to include genes coding for a virB4 homolog and a relaxase, along with a complement of *tra* genes similar to that found on F-plasmids. Based on this analysis the plasmid should be classified as conjugative (or at least mobilizable) [79].

## Additional files

Additional file 1: **Tables S1-S8.** Informational tables for GDPD-like SMaseD/PLD sequences considered in this study. (XLS 140 kb)

Additional file 2: **Figure S1.** Sequence alignment of all GDPD-like SMaseD/PLD domains used for phylogenetic tree construction. Sequence names include an abbreviation for genus and species (e.g. Tetr_ur for *Tetranychus urticae*), preceded by a number to account for multiple homologs from a given species, and followed by a protein or nucleotide identifier for the database source of the sequence. (PDF 4708 kb)

Additional file 3: **Figure S2.** Sequence alignment of cysteine-rich C-terminal domains found among Aquatic clade homologs. Note the presence of 10 perfectly conserved cysteine residues per domain, along with a glycine-rich motif and conserved tyrosine at the C-terminal end (asterisks). Sequence names include an abbreviation for genus and species, preceded by a number to account for multiple homologs from a given species and a letter to account for multiple domains within a homolog, e.g. “b1_Eurh_ex” denotes the 2nd cysteine-rich C-terminal domain found in homolog 1 from *Eurhomalea exalbida.* The sequence names is also tagged with a protein or nucleotide identifier for the database source of the sequence. (PDF 375 kb)

## Abbreviations

BLAST: Basic Local Alignment Search Tool
cPA: Cyclic phosphodiesterase
DNA: Deoxyribonucleic acid
FFAS: Fold and Function Assignment
GDPD: Glycerophosphoryl diester phosphodiester phosphodiesterase
LGT: Lateral gene transfer
mRNA: Messenger ribonucleic acid
NCBI: National Center for Biotechnology Information
ORF: Open reading frame
PDB: Protein Data Bank
PLD: Phospholipase D
SMase: Sphingomyelinase
SRA: Sequence read archive
VAST: Vector Alignment Search Tool
