# Supplementary material for: Evolutionary dynamics of origin and loss in the deep history of phospholipase D toxin genes
Source: BMC Evol Biol. 2018 Dec 18;18:194. doi: 10.1186/s12862-018-1302-2 (PMC6299612; doi:10.1186/s12862-018-1302-2)

Figure S1. Sequence alignment of all GDPD-like SMaseD/PLD domains used for phylogenetic tree construction. Sequence names include an abbreviation for genus and species (e.g. Tetr_ur for *Tetranychus urticae*), preceded by a number to account for multiple homologs from a given species, and followed by a protein or nucleotide identifier for the database source of the sequence.

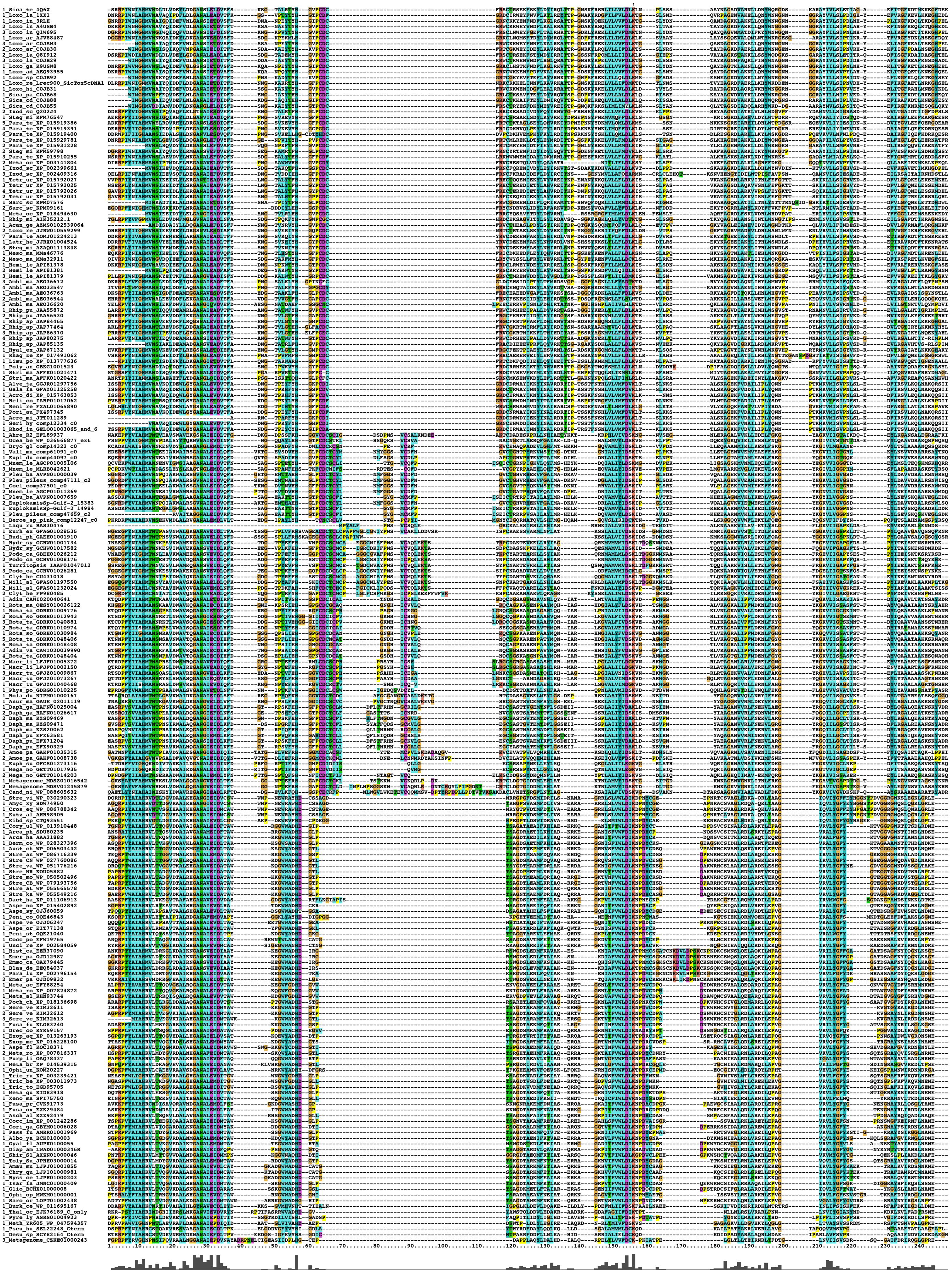

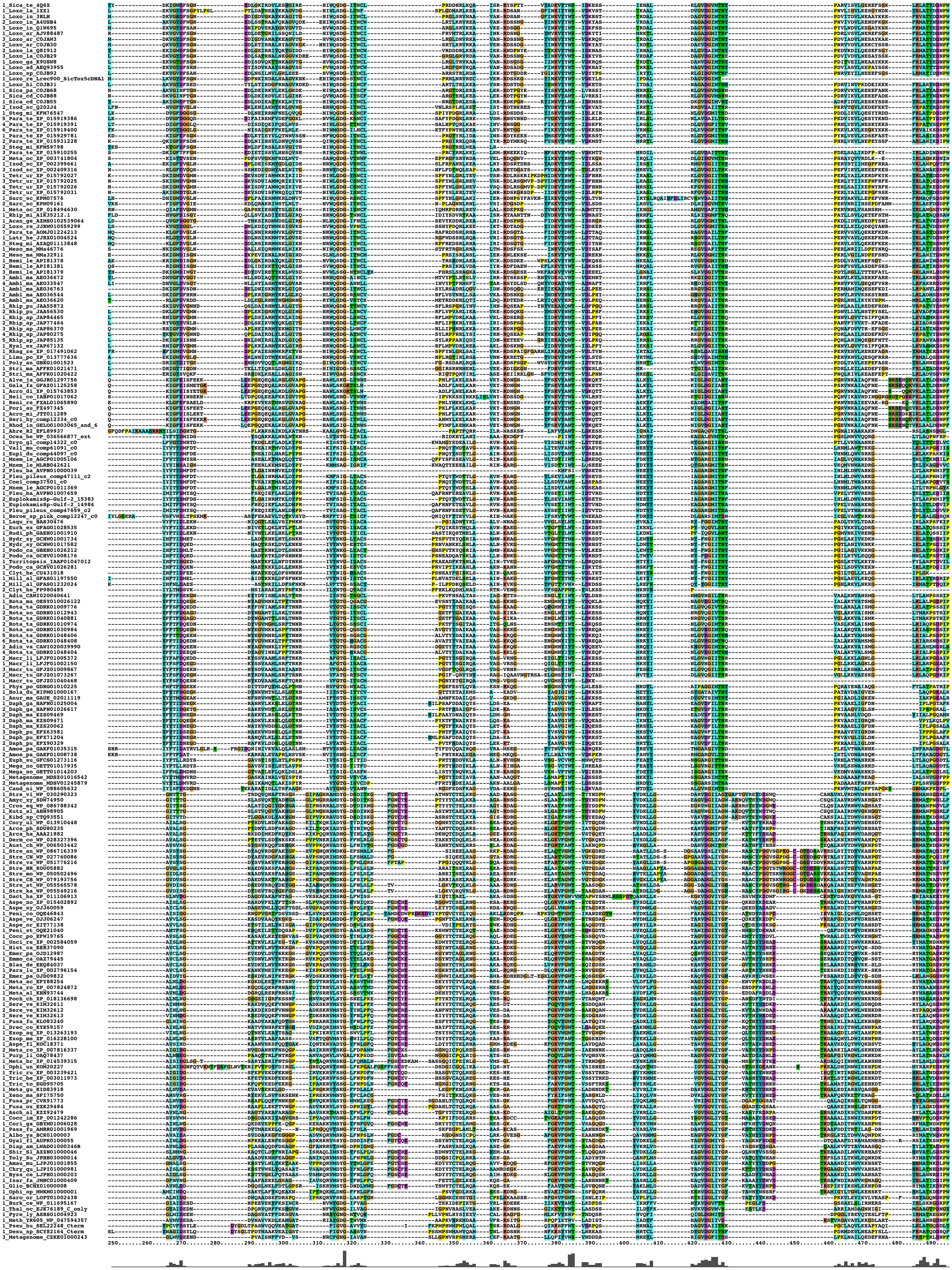

Supplement: Supplementary file 2 — Figure S1. Sequence alignment of all GDPD-like SMaseD/PLD domains used for phylogenetic tree construction. Sequence names include an abbreviation for genus and species (e.g. Tetr_ur for Tetranychus urticae), preceded by a number to account for multiple homologs from a given species, and followed by a protein or nucleotide identifier for the database source of the sequence. (PDF 4708 kb) [file 12862_2018_1302_MOESM2_ESM.pdf]
