# Supplementary material for: Evolutionary dynamics of origin and loss in the deep history of phospholipase D toxin genes
Source: BMC Evol Biol. 2018 Dec 18;18:194. doi: 10.1186/s12862-018-1302-2 (PMC6299612; doi:10.1186/s12862-018-1302-2)
Supplement: Supplementary file 3 — Figure S2. Sequence alignment of cysteine-rich C-terminal domains found among Aquatic clade homologs. Note the presence of 10 perfectly conserved cysteine residues per domain, along with a glycine-rich motif and conserved tyrosine at the C-terminal end (asterisks). Sequence names include an abbreviation for genus and species, preceded by a number to account for multiple homologs from a given species and a letter to account for multiple domains within a homolog, e.g. “b1_Eurh_ex” denotes the 2nd cysteine-rich C-terminal domain found in homolog 1 from Eurhomalea exalbida. The sequence names is also tagged with a protein or nucleotide identifier for the database source of the sequence. (PDF 375 kb) [file 12862_2018_1302_MOESM3_ESM.pdf]

Figure S2. Sequence alignment of cysteine-rich C-terminal domains found among Aquatic clade homologs. Note the presence of 10 perfectly conserved cysteine residues per domain, along with a glycine-rich motif and conserved tyrosine at the C-terminal end (asterisks). Sequence names include an abbreviation for genus and species, preceded by a number to account for multiple homologs from a given species and a letter to account for multiple domains within a homolog, e.g. "b1_Eurh_ex" denotes the 2nd cysteine-rich C-terminal domain found in homolog 1 from *Eurhomalea exalbida.* The sequence names is also tagged with a protein or nucleotide identifier for the database source of the sequence.

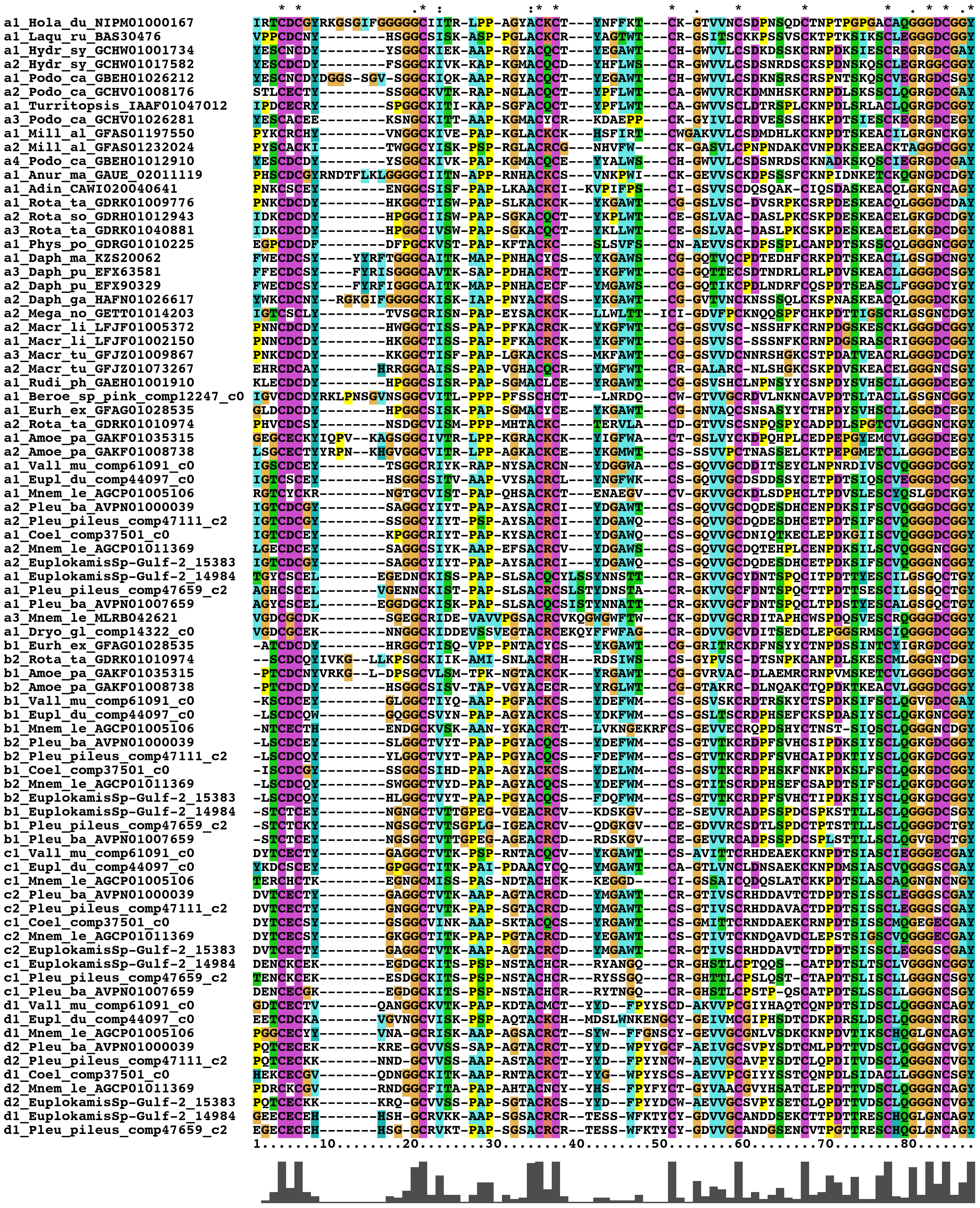

a1_Hola_du_NIPM01000167 IRTCDCGYRKGSGIFGGGGGCIITR-LPP-AGYACKCT---YNFFKT---CK-GTVVNCSDPNSQDCTNPTPGPGACAQGGGDCGGY
a1_Laqu_ru_BAS30476 VPPCDCNY--------HSGGCSISK-ASP-PGLACKCR---YAGTWT---CR-GSITSCKKPSSVSCKTPTKSIKSCLEGGGDCGGY
a1_Hydr_sy_GCHW01001734 YESCNCDY--------YSGGCKIEK-AAP-RGYACQCT---YEGAWT---CH-GWVVLCSDKDSSKCRNPDKSIESCREGRGDCGAY
a2_Hydr_sy_GCHW01017582 YESCDCDY--------FSGGCKIVK-KAP-KGMACQCD---YHFLWS---CR-GWVVLCSDSNRDSCKSPDNSKQSCLEGRGGCGGY
a1_Podo_ca_GBEH01026212 YESCNCDYDGGS-SGV-SGGCKIQK-AAP-RGYACQCT---YEGAWT---CH-GWVVLCSDKNSRSCRSPNTSKQSCVEGRGDCSGY
a2_Podo_ca_GCHV01008176 STLCECTY--------SSGGCKVTK-RAP-NGLACQCT---YPFLWT---CA-GWVVLCRKDMNHSKCRNPDLSKSSCLQGRGDCGAY
a1_Turritopsis_IAAF01047012 IPDCECRY--------SPGGCKITI-KAP-SGFACQCT---YPFLWT---CA-GWVVSCLDTRSPLCKNPDLSRLACLQGRGDCGGY
a3_Podo_ca_GCHV01026281 YESCACEE--------KSNGCKITT-AAP-KGMACYCR---KDAEPP---CK-GYIVLCRDVESSSCHKPDTSIESCKEGRGDCEGY
a1_Mill_al_GFAS01197550 PYKCRCHY--------VNGGCKIVE-PAP-KGLACKCK---HSFIRT---CWGAKVVLCSDMDHLKCKNPDTSKEACILGRGNCKGY
a2_Mill_al_GFAS01232024 PYSCACKI--------TWGGCYISK-PSP-RGLACRCG---NHVFW----CK-GASVLCPNPNDAKCVNPDKSEEACKTAGGDCGGY
a4_Podo_ca_GBEH01012910 YESCDCDY--------YSGGCKIVK-PAP-KGMACQCE---YYALWS---CH-GWVVLCSDSNRDSCKNADKSKQSCIEGRGDCGAY
a1_Anur_ma_GAUE_02011119 PHSCDCGYRNDTFLKLGGGGCIITN-APP-RNHACKCS---VNKPWI---CK-GEVVSCKDPSSSFCKNPIDNKETCKQGNGDCDGY
a1_Adin_CAWI020040641 PNKCSCEY--------ENGGCSISF-PAP-LKAACKCI--KVPIFPS---CI-GSVVSCDQSQAK-CIQSDASKEACQLGKGNCAGY
a1_Rota_ta_GDRK01009776 PNKCDCDY--------HKGGCTISW-PAP-SLKACKCK---YKGAWT---CG-GSLVSC-DVSRPKCSRPDESKEACQLGGGDCDAY
a2_Rota_so_GDRH01012943 IDKCDCDY--------HPGGCIISW-PAP-SGKACQCT---YKPLWT---CE-GSLVAC-DASLPKCSKPDESKEACELGKGDCDGY
a3_Rota_ta_GDRK01040881 IDKCDCDY--------HPGGCIVSW-PAP-SGKACQCT---YKLLWT---CE-GSLVAC-DASLPKCSKPDESKEACELGKGDCDGY
a1_Phys_po_GDRG01010225 EGPCDCDF--------DFPGCKVST-PAP-KFTACKC---SLSVFS---CN-AEVVSCKDPSSPLCANPDTSKSSCQLGGGNCGGY
a1_Daph_ma_KZS20062 FWECDCSY-----YYRFTGGGCAITK-MAP-PNHACYCS---YKGAWS---CG-GQTVQCPDTEDHFCRKPDTSKEACLLGSGDCNGY
a3_Daph_pu_EFX63581 FFECDCSY----FYRISGGGCAVTK-SAP-PDHACRCT---YKGFWT---CG-GQTTECSDTNDRLCRLPDVSKEACLLGGGDCSGY
a2_Daph_pu_EFX90329 FWECDCSY----FYRFIGGGCAITK-MAP-PNHACECF---YMGAWS---CG-GQTIKCPDLNDRFCQSPDTSEASCLFGGGDCYGY
a2_Daph_ga_HAFN01026617 YWKCDCNY--RGKGIFGGGGCKISK-IAP-PNYACKCS---YKGAWT---CG-GVTVNCKNSSSQLCKSPNASKEACQLGGGDCKGY
a2_Mega_no_GETT01014203 IGTCSCLY--------TVSGCRISN-PAP-EYSACKCK---LLWLTT--ICI-GDVFPCKNQQSPFCHKPDTTIGSCRLGSGNCDGY
a2_Macr_li_LFJF01005372 PNNCDCDY--------HWGGCTISS-PAP-PFKACRCK---YKGFWT---CG-GSVVSC-NSSHFKCRNPDGSKESCKLGGGDCGGY
a1_Macr_li_LFJF01002150 PNNCDCDY--------HKGGCTISS-PAP-PFKACRCK---YKGFWT---CG-GSVVSC-SSSNFKCRNPDGSRASCRIGGGDCGGY
a3_Macr_tu_GFJZ01009867 PNKCDCDY--------KKGGCTISF-PAP-LGKACKCS---MKFAWT---CG-GSVVDCNNRSHGKCSTPDATVEACRLGGGDCDGY
a2_Macr_tu_GFJZ01073267 EHRCDCAY-------HRRGGCAISS-PAP-VGHACQCR---YMGFWT---CR-GALARC-NLSHGKCSKPDVSKEACELGGGNCSGY
a1_Rudi_ph_GAEH01001910 KLECDCDY--------HPGGCSISR-PAP-SGMACLCE---YRGAWT---CG-GSVSHCLNPNSYYCSNPDYSVHSCLLGGGDCEGY
a1_Beroe_sp_pink_comp12247_c0 IGVCDCDYRKLPNSGVNSGGCVITL-PPP-PFSSCHCT----LNRDQ---CW-GTVVGCRDVLNKNCAVPDTSLTACLLGSGNCGGY
a1_Eurh_ex_GFAG01028535 GLDCDCDY--------HPGGCSISK-PAP-SGMACYCE---YKGAWT---CG-GNVAQCSNSASYYCTHPDYSVHSCLLGGGDCEGY
a2_Rota_ta_GDRK01010974 PHVCDCSY--------NSDGCVISM-PPP-MHTACKC----TERVLA---CD-GTVVSCSNPQSPYCADPDLSPGTCVLGGGNCKGY
a1_Amoe_pa_GAKF01035315 GEGCECKYIQPV--KAGSGGCIVTR-LPP-KGRACKCK---YIGFWA---CT-GSLVYCKDPQHPLCEDPEPGYEMCVLGGGDCEGY
a2_Amoe_pa_GAKF01008738 LSGCECTYYRPN--KHGVGGCVITR-LPP-AGKACKCE---YKGMWT---CS-SSVVPCTNASSELCKTPEPGMETCLLGGGDCEGY
a1_Vall_mu_comp61091_c0 IGSCDCEY--------TSGGCRIYK-RAP-NYSACRCN---YDGGWA---CS-GQVVGCDDITSEYCLNPNRDIVSCVQGGGDCEGY
a1_Eupl_du_comp44097_c0 IGTCSCEY--------HSGGCSITV-AAP-VYSACRCL---YNGFWK---CS-GQVVGCDSDDSSYCETPDTSIQSCVEGGGDCGGY
a1_Mnem_le_AGCP01005106 RGTCYCKR--------NGTGCVIST-PAP-QHSACKCT---ENAEGV---CV-GKVVGCKDLSDPHCLTPDVSLESCYQSLGDCKGY
a2_Pleu_ba_AVPN01000039 IGTCDCGY--------SAGGCYIYT-PAP-AYSACRCI---YDGAWT---CS-GQVVGCDQDESDHCENPDKSIFSCVQGGGDCGGY
a2_Pleu_pileus_comp47111_c2 IGTCDCGY--------SSGGCYIYT-PSP-AYSACRCI---YDGAWQ---CS-GQVVGCDQDESDHCETPDTSIFSCVQGGGDCGGY
a1_Coel_comp37501_c0 IGTCDCEY--------KPGGCRIYT-PAP-KYSACRCI---YDGAWQ---CS-GQVVGCDNIQTKECLEPDKGIISCVQGGGDCGGY
a2_Mnem_le_AGCP01011369 LGECDCEY--------SAGGCSIYK-AAP-EFSACRCV---YDGAWS---CS-GQVVGCDQTEHPLCENPDKSILSCVQGGGNCGGY
a2_EuplokamisSp-Gulf-2_15383 IGTCDCGY--------SAGGCYIYT-PAP-AYSACRCI---YDGAWQ---CS-GQVVRCKQDESDHCETPDKSIFSCVQGGGDCGGY
a1_EuplokamisSp-Gulf-2_14984 TGYCSCEL-------EGEDNCKISS-PAP-SLSACQCYLSSYNNSTT---CR-GKVVGCYDNTSPQCITPDTTYESCILGSGQCTGY
a1_Pleu_pileus_comp47659_c2 AGHCSCEL-------VGENNCKIST-PAP-SLSACRCSLSTYDNSTA---CR-GKVVGCFDNTSPQCTTPDTTSESCILGSGQCTGY
a1_Pleu_ba_AVPN01007659 AGYCSCEL-------EGGDGCKISK-PAP-SLSACQCSISTYNNATT---CR-GKVVGCFDNTSPQCLTPDSTYESCALGSGQCTGY
a3_Mnem_le_MLRB042621 VGDCGCDK--------SGEGCRIDE-VAVVPGSACRCVKQGWGWFTW---CK-GDVVGCRDITAPHCWSPDQSVESCRQGGGDCGGY
a1_Dryo_gl_comp14322_c0 VGDCGCEK--------NNGGCKIDDEVSSVEGTACRCEKQYFFWFAG---CR-GDVVGCVDITSEDCLEPGGSRMSCIQGGGDCEGY
b1_Eurh_ex_GFAG01028535 -ATCDCDY--------HRGGCTISQ-VPP-PNTACYCS---YKGAWT---CG-GRITRCKDFNSYYCTNPDSSINTCYIGRGDCGGY
b2_Rota_ta_GDRK01010974 --SCDCQYIVKG--LLKPSGCKIIK-AMI-SNLACRCH---RDSIWS---CS-GYPVSC-DTSNPKCANPDLSKESCMLGGGNCDGY
b1_Amoe_pa_GAKF01035315 -PTCDCNYVRKG--L-DPSGCVLSM-TPK-NGTACKCK---YRGAWT---CG-GVRVAC-DLAEMRCRNPVMSKETCVLGGGDCGGY
b2_Amoe_pa_GAKF01008738 -PTCDCDY--------HSGGCSISV-TAP-VGYACECR---YRGLWT---CG-GTAKRC-DLNQAKCTQPDKTKEACVLGGGDCEGY
b1_Vall_mu_comp61091_c0 -KSCDCEY--------GLGGCTIYQ-AAP-PGFACKCS---YDEFWM---CS-GSVSKCRDTRSKYCTSPDKSIFSCLQGVGDCGAY
b1_Eupl_du_comp44097_c0 -LSCDCQW--------GQGGCSVYN-PAP-AGYACKCS---YDKFWM---CS-GSLTSCRDPHSEFCKSPDASIYSCLQGKGNCGGY
b1_Mnem_le_AGCP01005106 -NTCECTH--------ENDGCKVSK-AAN-YGKACRCT---LVKNGEKRFCS-GEVVECRQPDSHYCTNST-SIQSCLQGGGNCDGY
b2_Pleu_ba_AVPN01000039 -LSCDCEY--------SLGGCTVYT-PAP-PGYACQCS---YDEFWM---CS-GTVTKCRDPFSVHCSIPDKSIYSCLQGKGDCGGY
b2_Pleu_pileus_comp47111_c2 -ISCDCQY--------YLGGCTVYT-PAP-PGYACQCS---FDEFWM---CS-GTVTKCRDPFSVHCAIPDKTIYSCLQGKGDCGGY
b1_Coel_comp37501_c0 -ISCDCGY--------SSGGCSIHD-PAP-AGYACKCS---YDELWM---CS-GSVTKCRDPHSKFCNKPDKSLFSCLQGKGDCGGY
b2_Mnem_le_AGCP01011369 -LSCDCQY--------SWGGCTVYD-PAP-AGYACQCS---YDEFWM---CS-GTITSCRDPHSEFCSAPDTSIFSCLQGKGDCGGY
b2_EuplokamisSp-Gulf-2_15383 -LSCDCQY--------HLGGCTVYT-PAP-PGYACQCS---FDQFWM---CS-GTVTKCRDPFSVHCTIPDKSIYSCLQGKGDCGGY
b1_EuplokamisSp-Gulf-2_14984 -STCTCEY--------NGNGCTVTTGPEG-VGEACRCV---KDSKGV---CE-SEVVRCADPSSPDCSPKSTTLLSCLQGGGDCSGY
b1_Pleu_pileus_comp47659_c2 -STCTCKY--------NGSGCTVTSGPLG-IGEACRCV---QDGKGV---CE-GDVVRCSDTLSPDCTPTSTTLLSCLQGKGDCTGY
b1_Pleu_ba_AVPN01007659 -STCTCEY--------NGSGCTVTTGPEG-AGEACRCD---RDSKGV---CE-GEVVRCADPSSPDCSPLSTTLLSCLQGVGDCTGY
c1_Vall_mu_comp61091_c0 DYTCECTY--------GAGGCTVTK-PSP-RNTACQCV---YKGAWT---CS-AVITTCRHDEAEKCKNPDTSIASCIEGGGECGAY
c1_Eupl_du_comp44097_c0 YKDCSCEY--------GPGGCTITK-PAI-PDAACYCQ---YMGAWT---CA-GTLVNCLDNSAEKCKNPDMSIASCVQGGGSCGAY
c1_Mnem_le_AGCP01005106 TERCHCTK--------EGNGCMISS-PAS-NDTACHCK---KEGGD----CI-GSSAICQDQSLATCKKPDTSLASCAQGNGNCNGY
c2_Pleu_ba_AVPN01000039 DVTCECTY--------GAGGCTVTK-AAP-AGTACRCD---YMGAWT---CR-GTIVSCRHDDAVTCTDPDTSISSCLEGGGSCGAY
c2_Pleu_pileus_comp47111_c2 DVTCECTY--------GNGGCTVTK-PAP-SGTACRCD---YMGAWT---CS-GTIVSCRHDDAVACTDPDTSISSCLEGGGSCGAY
c1_Coel_comp37501_c0 DYTCECSY--------GSGGCVINK-AAP-SKTACQCS---YRGAWT---CS-GMITTCRNDDAEKCRNPDTSISSCMQGEGECGAY
c2_Mnem_le_AGCP01011369 DYTCECSY--------GKGGCTITK-RAP-PGTACRCD---YEGAWT---CS-GTIVTCKNDQAVDCLEPSTSIGSCVQGGGECGAY
c2_EuplokamisSp-Gulf-2_15383 DVTCECTY--------GAGGCTVTK-AAP-AGTACRCD---YMGAWT---CR-GTIVSCRHDDAVTCTDPDTSISSCLEGGGSCGAY
c1_EuplokamisSp-Gulf-2_14984 DENCKCEK--------EGDGCKITS-PSP-NSTACHCR---RYANGQ---CR-GHSTLCPTQQS--CATPDTSLTSCLVGGGNCGGY
c1_Pleu_pileus_comp47659_c2 TENCKCEK--------ESDGCKITS-PSP-NSTACHCR---RYSSGQ---CR-GHTTLCPSLQST-CTAPDTSLISCLLGGGNCSGY
c1_Pleu_ba_AVPN01007659 DENCECGK--------EGDGCKITS-PSP-NSTACHCR---RYTNGQ---CR-GHSTLCPSTP-QSCATPDTSLSSCLLGGGNCSGY
d1_Vall_mu_comp61091_c0 GDTCECTV-------QANGGCKVTK-PAP-KDTACMCT--YYD--FPYYSCD-AKVVPCGIYHAQTCQNPDTSIDSCLQGGGNCAGY
d1_Eupl_du_comp44097_c0 EETCDCKA-------VGVNGCVISK-PSP-AQTACKCH--MDSLWNKENGCY-GEIVMCGIPHSDTCDKPDRSLDSCLQGGGNCRGY
d1_Mnem_le_AGCP01005106 PGGCECYY-------VNA-GCRISK-AAP-AGSACRCT--SYW--FFGNSCY-GEVVGCGNLVSDKCKNPDVTIKSCHQGLGNCAGY
d2_Pleu_ba_AVPN01000039 PQTCECEK-------KRE-GCVVSS-SAP-AGTACRCT--YYD--WPYYGCF-AEIVGCSVPYSDTCMLPDTTVDSCLQGGGNCVGY
d2_Pleu_pileus_comp47111_c2 PQTCECKK-------NND-GCVVSS-AAP-ASTACRCT--YYD--FPYYNCW-AEVVGCAVPYSDTCLQPDITVDSCLQGGGNCVGY
d1_Coel_comp37501_c0 HEKCECGV-------QDNGGCKITK-AAP-RNTACKCT--YYG--WPYYSCS-AEVVPCGIYYSSTCQNPDLSIDACLLGGGNCSGY
d2_Mnem_le_AGCP01011369 PDRCKCGV-------RNDGGCFITA-PAP-AHTACNCY--YHS--FPYFYCT-GYVAACGVYHSATCLEPDTTVDSCLQGGGNCAGY
d2_EuplokamisSp-Gulf-2_15383 PQTCECKK-------KRQ-GCVVSS-PAP-SGTACRCS--YYD--FPYYDCW-AEVVGCSVPYSETCLQPDTTVDSCLQGGGNCVGY
d1_EuplokamisSp-Gulf-2_14984 GEECECEH-------HSH-GCRVKK-AAP-SGSACRCR--TESS-WFKTYCY-GDVVGCANDDSEKCTTPDTTRESCHQGLGNCAGY
d1_Pleu_pileus_comp47659_c2 EGECECEH-------HSG-GCRVKT-PAP-SGSACRCR--TESS-WFKTYCY-GDVVGCANDGSENCVTPGTTRESCHQGLGNCAGY
1.......10........20........30........40........50........60........70........80.......
